# Liquid-liquid phase separation throws novel insights into treatment strategies for skin cutaneous melanoma

**DOI:** 10.1186/s12885-023-10847-w

**Published:** 2023-05-01

**Authors:** Jianlan Liu, Shengbin Pei, Pengpeng Zhang, Keyu Jiang, Binlin Luo, Zuoqiong Hou, Gang Yao, Jian Tang

**Affiliations:** 1 Department of Plastic and Burns Surgery, The First Affiliated Hospital of Nanjing Medical University, Nanjing, China; 2 Department of Breast Surgery, The First Affiliated Hospital of Nanjing Medical University, Nanjing, China; 3 Department of Thoracic Surgery, The First Affiliated Hospital of Nanjing Medical University, Nanjing, China

**Keywords:** LLPS, Melanoma, TROAP, Tumor microenvironment, Immunotherapy

## Abstract

**Background:**

In recent years, there has been growing evidence indicating a relationship between liquid–liquid phase separation (LLPS) and cancer development. However, to date, the clinical significance of LLPS in skin cutaneous melanoma (SKCM, hereafter referred to as melanoma) remains to be elucidated. In the current study, the impact of LLPS-related genes on melanoma prognosis has been explored.

**Methods:**

LLPS-related genes were retrieved from the DrLLPS database. The prognostic feature for LLPS in melanoma was developed in The Cancer Genome Atlas (TCGA) dataset and verified in the GSE65904 cohort. Based on risk scores, melanoma patients were categorized into high- and low-risk groups. Thereafter, the differences in clinicopathological correlation, functional enrichment, immune landscape, tumor mutational burden, and impact of immunotherapy between the two groups were investigated. Finally, the role of key gene TROAP in melanoma was validated by in vitro and in vivo experiments.

**Results:**

The LLPS-related gene signature was developed based on MLKL, PARVA, PKP1, PSME1, RNF114, and TROAP. The risk score was a crucial independent prognostic factor for melanoma and patients with high-risk scores were related to a worse prognosis. Approximately, all immune-relevant characteristics, such as immune cell infiltration and immune scores, were extremely evident in patients with low-risk scores. The findings from the in vitro and in vivo experiments indicated that the viability, proliferation, and invasion ability of melanoma cells were drastically decreased after the knockdown of TROAP.

**Conclusion:**

Our gene signature can independently predict the survival of melanoma patients. It provides a basis for the exploration of the relationship between LLPS and melanoma and can offer a fresh perspective on the clinical diagnosis and treatment of the disease.

**Supplementary Information:**

The online version contains supplementary material available at 10.1186/s12885-023-10847-w.

## Introduction

Skin cutaneous melanoma (SKCM, hereafter referred to as melanoma) is a deadly form of skin cancer with a growing global prevalence [1]. As per the latest statistics available in 2020, 325,000 new cases of melanoma and 57,000 deaths from melanoma were reported [2]. If this rate continues, the melanoma burden is estimated to amount to 510,000 new cases (a roughly 50% increase) and 96,000 deaths (a 68% increase) by 2040 [3]. Currently, surgical resection is the primary treatment for early melanoma patients. However, the prognosis of patients, in particular of those with distant metastasis or recurrence, remains despairing with a 5-year survival rate of around 27% [4, 5]. Immune checkpoint inhibitors (ICIs) have achieved remarkable success in tumor therapy, especially in melanoma treatment [6]. Currently, BRAFV600E/K inhibitors represent the ideal first-line treatment for patients with BRAFV600E/K mutation-positive unresectable or metastatic melanoma [7, 8]. Meanwhile, developing inhibitors targeting MEK1/2 kinases has yielded the non-ATP-competitive allosteric inhibitors which brought hope for non-BRAF-mutant melanoma cases [9]. In the COMBI-d trial and the COMBI-v trial, patients with previously untreated BRAF V600E or V600K mutant unresectable or metastatic melanoma underwent randomization to receive dabrafenib plus trametinib. The result demonstrated that patients who were treated with the combination of dabrafenib and trametinib had significantly longer overall and progression-free survival than those treated with vemurafenib or dabrafenib alone. The clinical application of dabrafenib plus trametinib provides long-term benefit and brings confidence for metastatic melanoma patients [10–12]. Nevertheless, a low immune response rate and the inevitable drug resistance to treatment limit the number of patients benefiting from these novel therapies [13, 14]. Therefore, a novel predictive biomarker is essential to stratify which melanoma patients will benefit the most from each treatment.

Various intracellular processes are organized by liquid–liquid phase separation (LLPS) via the formation of membrane-less organelles called biomolecular condensates [15, 16]. Phase separation is the density-dependent separation of biomolecules from a homogeneous environment into two different phases (the condensed and dilute phases) [17]. Aberrant LLPS is widely associated with several hallmarks of cancer, including sustained proliferative signaling, growth suppressor evasion, cell death resistance, telomere maintenance, and DNA damage repair [18]. Previous studies suggested that LLPS-related gene signature significantly predicted prognosis in lower-grade glioma [19], ovarian cancer [20], and digestive system malignancies [21], and provided useful references for precise tumor management. Hence, we hypothesized that LLPS-related genes may be potential prognostic markers that could contribute to the further classification of melanoma and the development of personalized medicine to treat the disease.

A comprehensive investigation of LLPS-linked genes and their association with melanoma was conducted based on the cancer genome atlas (TCGA) dataset. Eventually, a gene signature consisting of six LLPS-associated genes was identified, and the underlying functions and immunological status of modeling genes were studied through a variety of analyses. GSE65904 was selected to validate the reliability of the model. Importantly, the function of the key gene TROAP in melanoma was validated by in vitro experiments. Our research might facilitate the prediction of individualized prognosis and better treatment options for melanoma patients.

## Materials and Methods

### Melanoma samples and LLPS-related gene source

The RNA-sequence (FPKM normalized) and clinical information of skin cutaneous melanoma (SKCM) samples were collected from the publicly accessible database—TCGA (https://portal.gdc.cancer.gov/, accessed on 30 March 2022). Patients having less than 30 days of follow-up or incomplete data were eliminated from further analysis. The gene expression profiles of normal skin tissue were downloaded from the Genotype-Tissue Expression (GTEX, http://www.gtexportal.org, accessed on 30 March 2022). For external validation studies, the GSE65904 dataset was extracted from the Gene Expression Omnibus database (GEO, https://www.ncbi.nlm.nih.gov/geo/, accessed on 30 March 2022), containing gene expression profiles and clinical material from other 214 melanoma patients. The genes were reannotated using the Per script, and the Combat function in the “sva” package was used to eliminate the batch effects before data analysis. The LLPS-related genes were obtained from DrLLPS [22] (http://llps.biocuckoo.cn/, accessed on 30 March 2022), which is a comprehensive database with 150 scaffold proteins acting as LLPS drivers, 987 LLPS regulators, and 8148 potential client proteins that may be essential for membrane-less organelles (MLOs) formation. The LLPS-associated genes experimentally identified in *Homo sapiens* were retained, and 3598 of them were screened for subsequent studies.

### Construction of an LLPS-related gene signature

A univariate Cox analysis of the overall survival (OS) of TCGA cohorts was implemented to filter the LLPS-related genes with a prognostic capacity of *p* < 0.01. The least absolute shrinkage and selection operator (LASSO) Cox analysis method was employed to screen the alternative genes and establish prognostic features using the “glmnet” package. Melanoma patients were randomly divided into two groups by the “caret” package according to 1:1 (the training and testing set). The LLPS-related gene signature was constructed as per the formula: Risk score =$\sum_{\mathrm{i}=1}^{\mathrm{n}}\mathrm{expi}*\mathrm{\beta i}$ (where expi indicates the expression of each LLPS-related gene and $\mathrm{\beta i}$ represents the respective coefficient of the gene). The risk score of each patient was independently determined in the TCGA training and testing groups using the aforementioned equation. Patients with risk scores exceeding the median value in the training set were stratified into high- or low-risk groups. In addition, the gene signature was verified in the GEO cohorts, and the risk score was similarly calculated to that of the TCGA dataset.

### Evaluation of the robustness of prognostic gene signature

Initially, the t-distributed stochastic neighbor embedding (t-SNE) and principal component analysis (PCA) were used for dimensionality reduction. The reliability of model grouping was determined through visual inspection. In the training set, survival curves, risk curves, survival status plots, and heatmaps were constructed to reflect prognostic differences between high- and low-risk groups. To demonstrate the validity and accuracy of the LLPS-related gene signature, the area under the curve (AUC) for 1-, 3- and 5-year OS was calculated using the “timeROC” package. Additionally, the same previous investigations were also performed with the testing set, the entire TCGA set, and the GSE65904 set. Data from the testing set was employed for the internal validation, and that from GSE65904 was applied for the external validation. In addition, the associations between the expression levels of candidate genes comprising the model and melanoma survival as well as clinical characteristics were analyzed.

### Identification of independent prognostic parameters in melanoma

To determine whether the gene signature was an independent predictor of prognosis, we applied both univariate and multivariate Cox regression analyses of clinicopathological features and risk scores of the entire TCGA group. Receiver operating characteristic (ROC) curves were plotted to indicate the predictive value of melanoma. The gene expression levels and clinical variables of the high- and low-risk groups were visualized with a heatmap. In addition, to provide a quantitative predictive scoring system for prognosis, a nomogram consisting of risk scores and clinical variables was developed to estimate the survival of melanoma patients. Calibration curves were plotted to determine whether practical survival was consistent with predicted survival.

### LLPS-based consensus clustering analysis

Melanoma patients were classified into diverse subtypes using the “ConsensusClusterPlus” package. The clustering results were visualized by PCA and t-SNE analysis. The prognosis between the different clusters was compared by the R package “survival” and “survminer”. Eventually, the correlation between the clusters and clinical variables was presented as a heatmap.

### Mutation patterns of LLPS-related genes in melanoma

To gain a comprehensive understanding of somatic mutations in melanoma patients, the “Masked Somatic Mutation” data of TCGA-SKCM was downloaded and visualized via the “maftools” package. Genetic alterations of six prognostic genes (MLKL, TROAP, PARVA, PKP1, PSME1, and RNF114) in melanoma patients were investigated in the cBioPortal database. Subsequently, tumor mutation burden (TMB) values corresponding to each melanoma patient generated from somatic mutation data were calculated. We assessed the correlation between the risk scores and TMB values. Next, melanoma patients were split into two groups based on their median TMB. The difference in survival probability between the two groups was compared. In addition, we assessed the correlations of TMB values with clinical features through the Wilcox test.

### Analysis of immune landscape and enrichment pathways in different risk groups

Four immune-related algorithms were used to investigate the differences in the immune landscape between various risk groups. The comparison of diverse immune cell activities or immune functions was performed by a single sample gene set enrichment analysis (ssGSEA). The relative abundance of tumor-infiltrating immune cells (TICs) in melanoma was determined using the CIBERSORT algorithm [23], which converts gene expression profiles into relative proportions of 22 TICs. The status of the tumor microenvironment (TME) can be evaluated by the ESTIMATE algorithm [24] based on the ratio of immune and stromal cells. The variance between immune checkpoint-associated genes in different risk populations was investigated using the rank-sum test. Immunophenoscore (IPS) primarily includes four components (effector cells, immunosuppressive cells, MHC molecules, and immune modulators) used to evaluate tumor immunogenicity [25]. A significant positive correlation exists between the immunophenoscore and tumor immunogenicity. The quantitative IPS ranges from 0 to 10, and a higher IPS for a patient indicates that the patient can benefit from immunotherapy [25]. Therefore, the IPS of melanoma patients in TCGA was calculated through The Cancer Immunome Atlas (TCIA, https://www.tcia.at, accessed on 30 March 2022). Eventually, to explore the potential mechanisms in two risk subgroups, the pathways enriched in two groups were determined by GSEA to identify and visualize LLPS-related representatives of the Kyoto Encyclopedia of Genes and Genomes (KEGG) pathways. In this analysis, the risk levels were considered as the phenotype, and the gene sets “c2.cp.kegg.v7.5.symbols.gmt” as the reference.

### Analysis of TROAP as a validating gene

The hazard ratio (HR) value of TROAP was the highest in the multivariate Cox analysis; hence, it was selected as the validation gene. The TROAP expression was first compared between the tumor and normal groups by combining normal tissue of the GTEX-skin dataset as a control. Thereafter, TROAP and other clinical characteristics were integrated into univariate and multivariate Cox regression analysis to determine whether TROAP could be an independent predictor. Meanwhile, the prognostic impact and relevance of immune cell infiltration of TROAP in melanoma were investigated. Finally, GSEA was performed to identify differences in functional pathways between the high- and low-TROAP groups, and TROAP expression was considered as a phenotypic label.

### Sample collection

Melanoma tissues and adjacent samples (*n* = 6) were surgically resected from melanoma patients (confirmed by postoperative pathology) treated in our hospital from April 2022 to August 2022. All these patients did not receive any treatment such as radiotherapy and chemotherapy before the operation. The resected tissues were then stored at –80 ℃. This study was authorized by the Ethics Committee of our hospital and conformed to the Declaration of Helsinki (No.2022-SR-465). Written informed consent was signed by the patients or their families.

### Cell culture and cell transfection

The human malignant melanoma cell lines A375 and WM-115 and human keratinocyte cells (HaCaT) utilized in this work were purchased from The Cell Bank of Type Culture Collection of the Chinese Academy of Sciences (https://www.cellbank.org.cn). A375 and WM-115 cells were cultured in DMEM medium (Gibco BRL, United States) containing 10% fetal bovine serum (FBS), (Gibco BRL, United States), 1% streptomycin/penicillin (Gibco; Thermo Fisher Scientific, Inc.) at 37 °C in 5% CO_2_. The cells were seeded in six-well plates and treated for 24 h with lentiviruses (Hanbio, Shanghai, China) including either shRNA sequences targeting TROAP or scrambled sequences (detailed lentivirus sequences for TROAP are available in Table S1) to construct TROAP knockdown and corresponding negative control cell lines respectively. Then, after 72 h of changing the medium, 1 μg/mL puromycin was added to kill uninfected cells. The efficiency of TROAP silencing in A375 and WM-115 cells was evaluated by real-time PCR analysis (RT-PCR).

### Quantitative real-time polymerase chain reaction

Total RNA was extracted from cells and tissues by TRIzol (15,596,018, Thermo, United States) following the manufacturer’s protocol and reverse transcribed to cDNA using PrimeScript™ RT reagent kit (R232-01, Vazyme, Nanjing, China). After adding primers and the SYBR Green qPCR Master Mix (Q111-02, Vazyme, Nanjing, China) to the newly synthesized cDNA and dissolving it in diethylpyrocarbonate (DEPC), QRT-PCR was performed on the LightCycler 480 Real-Time PCR System (Roche Diagnostics, South San Francisco, CA, United States). Each reaction was performed thrice. The data were analyzed by the 2^−∆∆Ct^ method and normalized using GAPDH as the internal reference. All primers were supplied by Tsingke Biotech (Tsingke, China), and detailed primer sequences are presented in Table S1.

## The Human Protein Atlas (HPA)

The HPA database (http://www.proteinatlas.org/) includes various protein expression data derived from immunohistochemistry (IHC) labelling of multiple malignancies. We acquired immunohistochemical staining data from the HPA database in order to investigate the protein expression levels of TROAP in melanoma tissues and surrounding tissues.

### CCK-8 assay

The cell counting kit-8 (CCK-8) assay was performed to explore the viability of melanoma cells. Briefly, cells were digested with trypsin and cultured in 96-well plates (3 × 10^3^ cells per well) overnight at 37 °C in 5% CO_2_. Thereafter, each well was incubated with 10 μL CCK-8 labeling reagent (A311-01, Vazyme, Nanjing, China) and 90 μL serum-free medium for 2 h in the dark at 37 ℃ before the assay. The absorbance of cells was measured at 450 nm wavelength on the enzyme-labeled meter (A33978, Thermo, United States) to analyze cell viability. Three independent replications were studied.

### Clone formation assay

Typically, clone formation assay is utilized to evaluate the proliferative capacity of cells. Five hundred transfected melanoma cells were cultured into 6-well plates. After two weeks, cell clones were visible to the naked eye. Next, the cells were rinsed twice with phosphate buffer saline (PBS) and fixed for 15 min in 4% paraformaldehyde. Thereafter, crystal violet (Solarbio, China) staining was performed for 20 min, the cells were dried at room temperature, and the number of cells per well was counted.

### EdU assay

EdU staining was performed using the EdU assay kit (Ribobio, Guangdong, China) following the manufacturer’s protocol. In brief, cells (2 × 10^4^ cells per well) were seeded into a 96-well plate and incubated with EdU in DMEM medium (50 μM). Following a 2-h incubation at 37 ℃ in 5% CO_2_, the cells were rinsed with PBS, and then soaked in 4% paraformaldehyde for 10 min at room temperature. Thereafter, they were permeabilized using 0.5% Triton-X-100, and stained with Apollo® fluorescent dye, according to the manufacturer’s protocol accompanying the Cell-Light EdU DNA cell proliferation kit from RiboBio. The cells were eventually observed under an inverted microscope to determine the percentage of EdU-positive cells.

### Wound healing assay

Transfected melanoma cells were seeded into 6-well plates and cultured under standard conditions until 95% confluent. Liner wounds were scratched with a sterile 200 μL pipette tip, non-adherent cells and debris were gently washed off with PBS. Next, cells were transferred to serum-free medium and incubated overnight. Eventually, photographs were taken at the same position at 0 h and 24 h, and the Image J software was used to measure the width of the scratches.

### Transwell assay

Transwell assay included cell migration and invasion experiments. The fundamental operations are described below: Treated cells (2 × 10^4^) were inserted into the upper chamber of 24-well plates and incubated for 24 h. The upper portion of the plate was either pre-coated with Matrigel solution (BD Biosciences, United States) to evaluate the invasive and migratory capabilities of the cells or was uncoated. The cells on the upper surface were removed. The remaining cells on the lower layer were fixed with 4% paraformaldehyde and stained with 0.1% crystal violet (Solarbio, China). The invasive cells were photographed and counted from three non-repetitive microscopy fields (× 100).

### Tumor xenograft model in vivo

All female five-week-old BALB/c nude mice used in this experiment were purchased from The Model Animal Research Center of Nanjing University (Nanjing, China). A375 cells (1 × 10^6 ^cells in 100 µL PBS volume) stably transfected with TROAP and control lentivirus, were subcutaneously inoculated into the flank of the mice independently for tumorigenicity tests. Tumor weights and volumes were measured with vernier calipers every five days, and the mice were euthanized after 20 days. The volume of the implanted tumor was calculated using the formula: volume = (width^2^ × length)/2. All animal experiments were performed according to the Committee on the Ethics of Animal Experiments guidelines at Nanjing Medical University.

### Statistical analysis

Bioinformatic data analysis was performed using R software (version 4.0.2), and experimental data analysis was conducted through GraphPad Prism 8.0 (GraphPad Software, USA). Clinicopathological characteristics were compared within the training and testing groups using the Chi-square test. The data were represented as the mean ± standard deviation (SD) of three independent experiments. Comparisons among the groups were performed using Student’s t-tests (for two groups) or one-way ANOVAs (for more than two groups) followed by Tukey’s test. *P < 0.05, **P < 0.01, ***P < 0.001.

## Results

### Development and evaluation of LLPS-related prognostic model based on the TCGA dataset

**Fig. 1:**
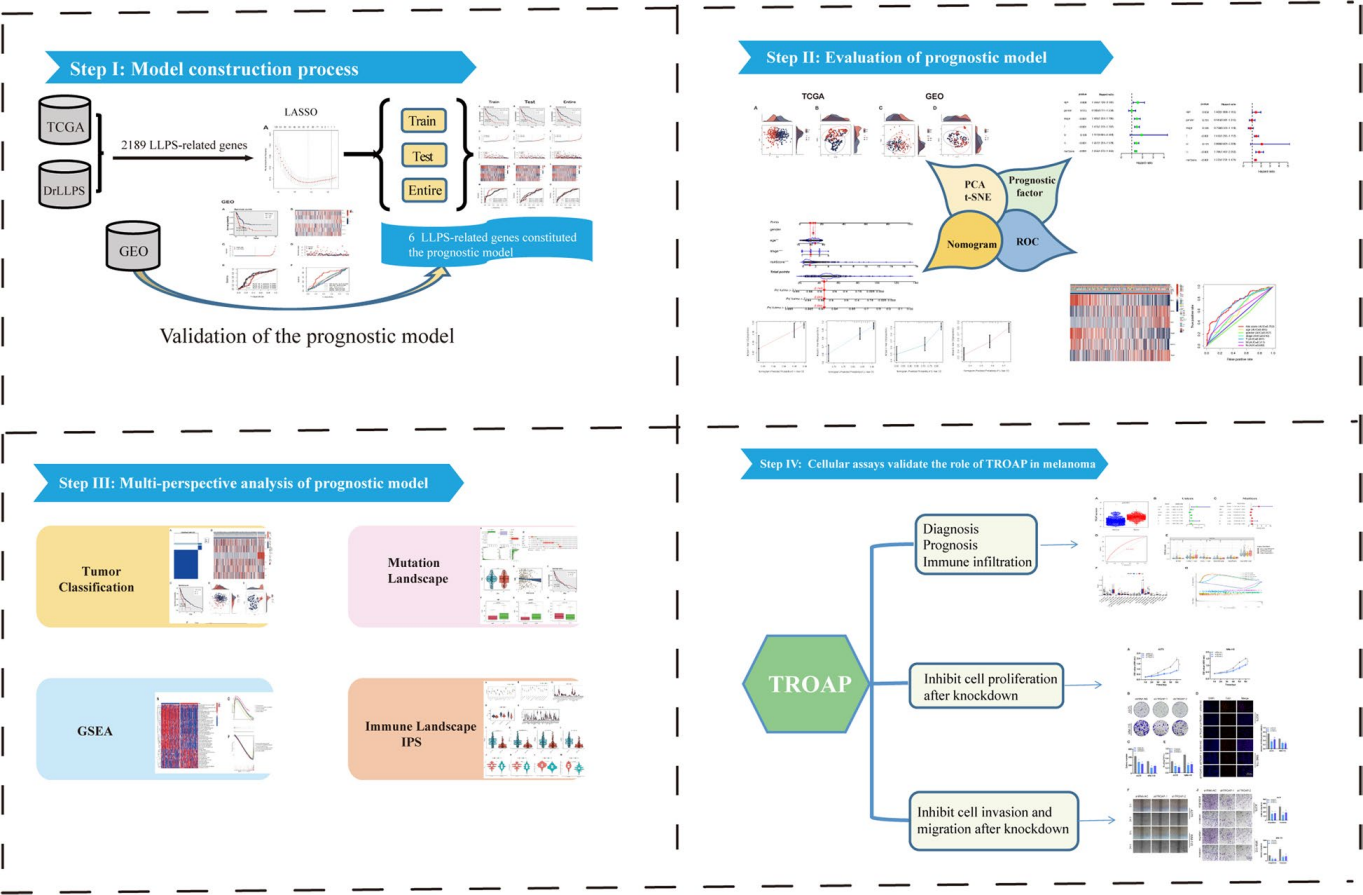
The workflow of the present study

**Fig. 2:**
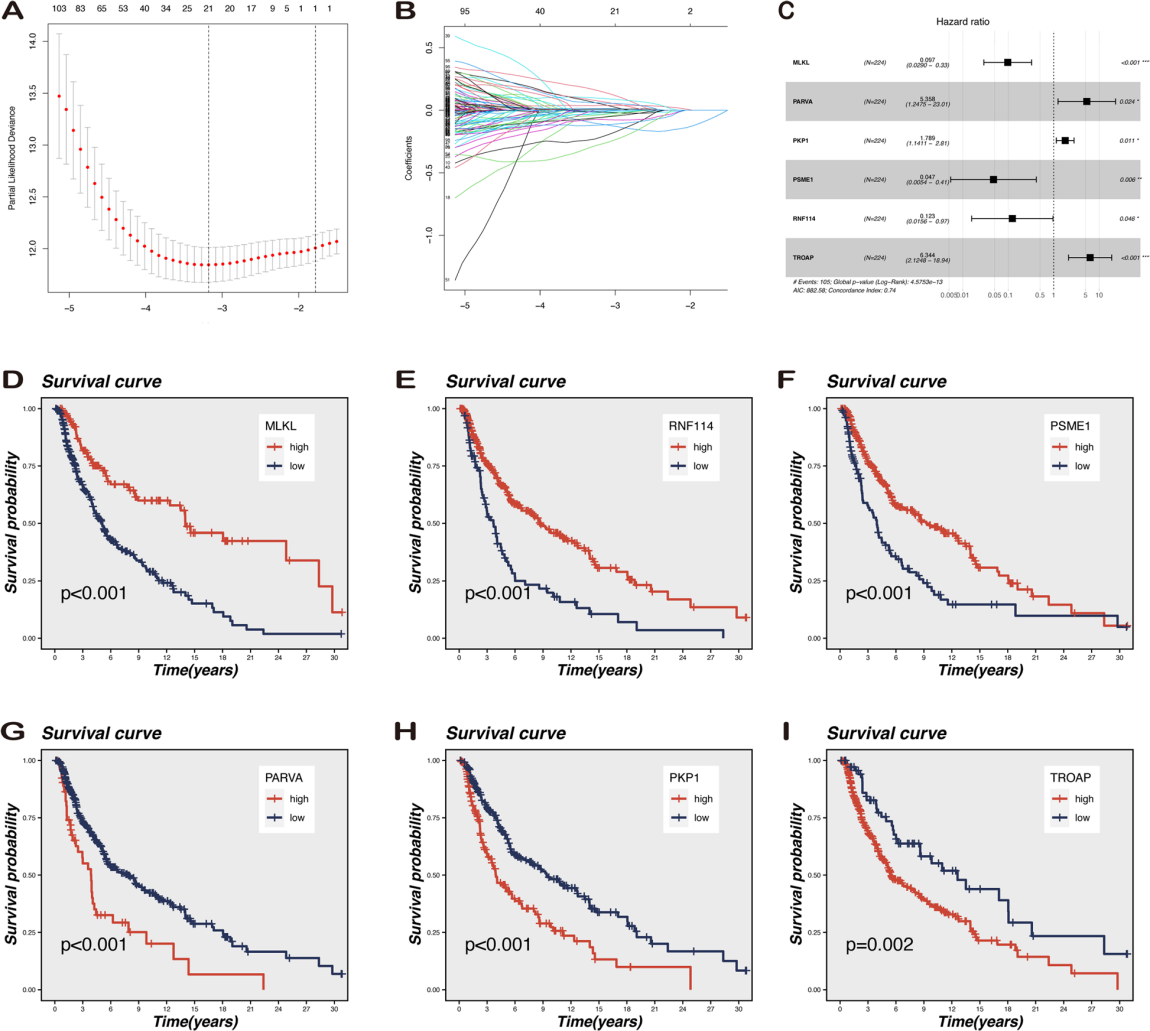
Construction of LLPS-related genes prognostic signature. **A**, **B** The optimal parameters were selected in the LASSO Cox regression analysis using tenfold cross-validation. **C** Multivariate Cox regression analysis demonstrated that six LLPS-related genes were independent prognostic factors for melanoma patients. **D**-**I** Survival analysis of six candidate genes. Poor prognosis was associated with low MLKL, RNF114, and PSME1, while high expression of PARVA, PKP1 and TROAP led to worse clinical outcomes. Note: *** *P* ≤ 0.001. ** *P* ≤ 0.01. * *P* ≤ 0.05

The detailed flow chart for the prognostic model construction is shown in Fig. 1. Furthermore, the clinicopathological characteristics of TCGA-SKCM patients are detailed in Table S2. In the table, no difference is observed in patient distribution between training and testing groups, which can allow for an independent analysis. The gene-expression data for 2189 LLPS-related genes is available in the TCGA (447 patients) and GEO (210 patients) cohorts. First, 164 survival-related genes are identified using univariate Cox regression analysis in the TCGA dataset (Table S3A). These 164 genes are incorporated into LASSO Cox regression analysis for further validation and selection of the best prognostic genes. Finally, six LLPS-related genes (MLKL, PARVA, PKP1, PSME1, RNF114, and TROAP) constitute the prognostic model and predicted clinical outcomes in melanoma patients (Fig. 2A, B). The corresponding risk score is obtained based on the coefficient of each cohort and the normalized expression of these six genes. The specific equation for the calculation is as follows: Risk score = MLKL*(-2.329) + PARVA*1.679 + PKP1*0.572 + PSME1*(-3.051) + RNF114*(-2.096) + TROAP*1.847 (Supplementary Table S3B). In Fig. 2C, MLKL, PSME1, and RNF114 are speculated to be protective genes, while PARVA, PKP1, and TROAP are probably risk genes. This conjecture was preliminarily confirmed in the subsequent gene survival analysis. The high expression levels of MLKL, RNF114, and PSME1 are associated with a better clinical outcome (Fig. 2D-F), whereas high PARVA, PKP1, and TROAP expression levels are associated with a worse prognosis (Fig. 2G–I).

### Evaluation of LLPS-related prognostic model based on the TCGA database

**Fig. 3:**
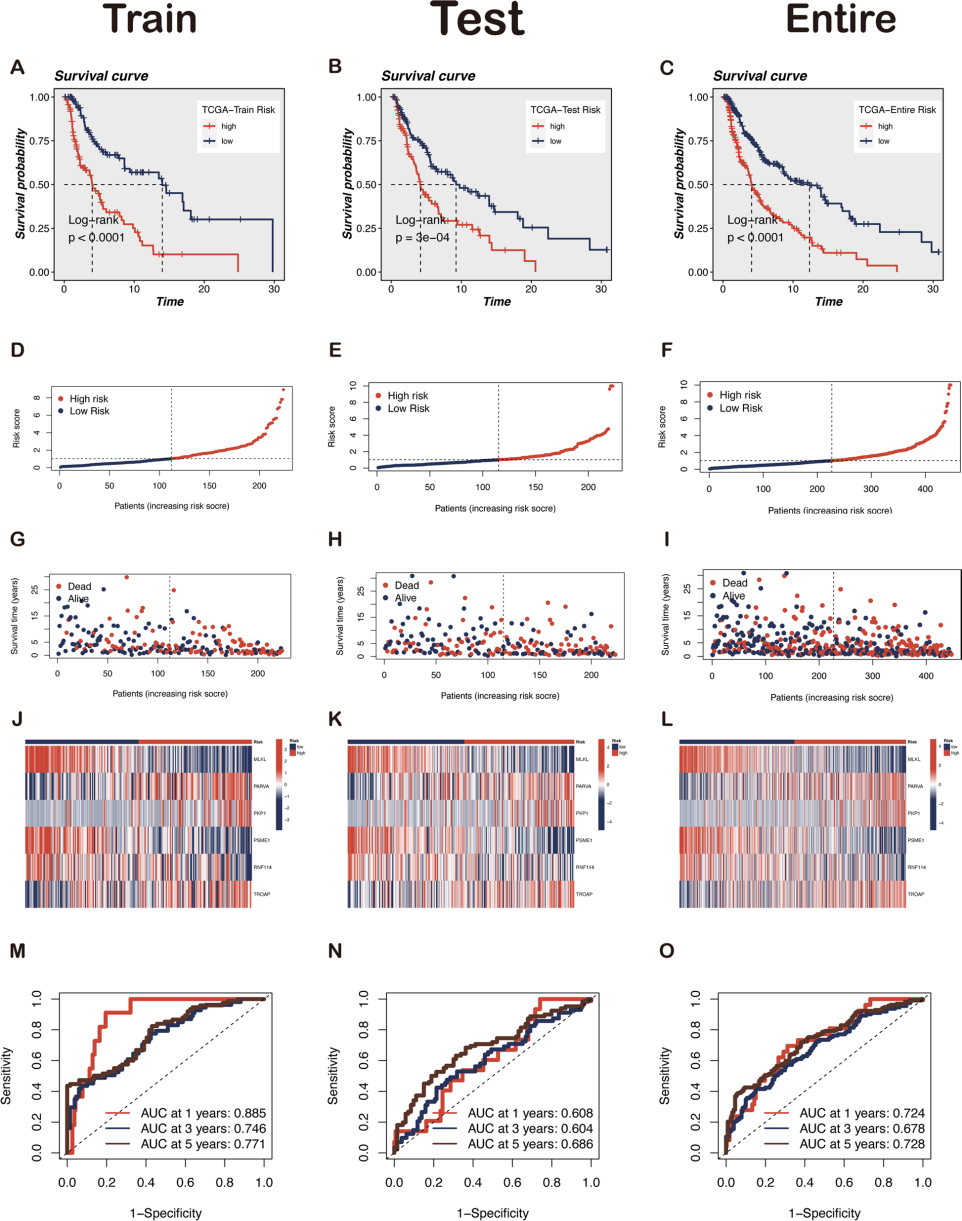
Comparison in stratified with risk signature of the six LLPS-associated genes in the TCGA cohort. **A**-**C** Overall survival was significantly different between patients with high and low risk scores. The distribution of the risk scores **D**-**F**, overall survival status **G**-**I**, and the expression of LLPS-related genes **J**-**L** among melanoma patients was shown (low-risk population: on the left side of the dotted line; high-risk population: on the right side of the dotted line; blue-black represents the number of survivors, and red represents the number of deaths. The risk from low to high reveals a rising tendency in deaths). **M**–**O** The ROC curves of the prognostic signature reflect the accuracy of the model in predicting patient survival at 1-, 3-, and 5 years

As shown in Fig. 3A–C, significant differences exist in survival rates between the high- and low-risk groups in the different cohorts (training set, testing set, and the entire set). The proportion of deaths increased with increasing risk scores, and clinical outcomes were worse in the high-risk group than that in the low-risk group (Fig. 3D–I). The distribution of risk scores and modeling gene expression levels in each cohort are shown in Fig. 3J–L. Notably, the LLPS-related gene signature achieves the AUC values of 0.885, 0.608, and 0.724 in the training, testing set, and entire TCGA sets, respectively, suggesting a substantially effective performance for OS prediction in melanoma (Fig. 3M–O). It is noteworthy that the PKP1 expression level is significantly linked to stage, T, and M and RNF114 expression level is correlated with T and stage. Furthermore, the TROAP expression level has a crucial influence on N classification (Figure S1).

### Validation of the prognostic value of risk score using the GEO database

**Fig. 4:**
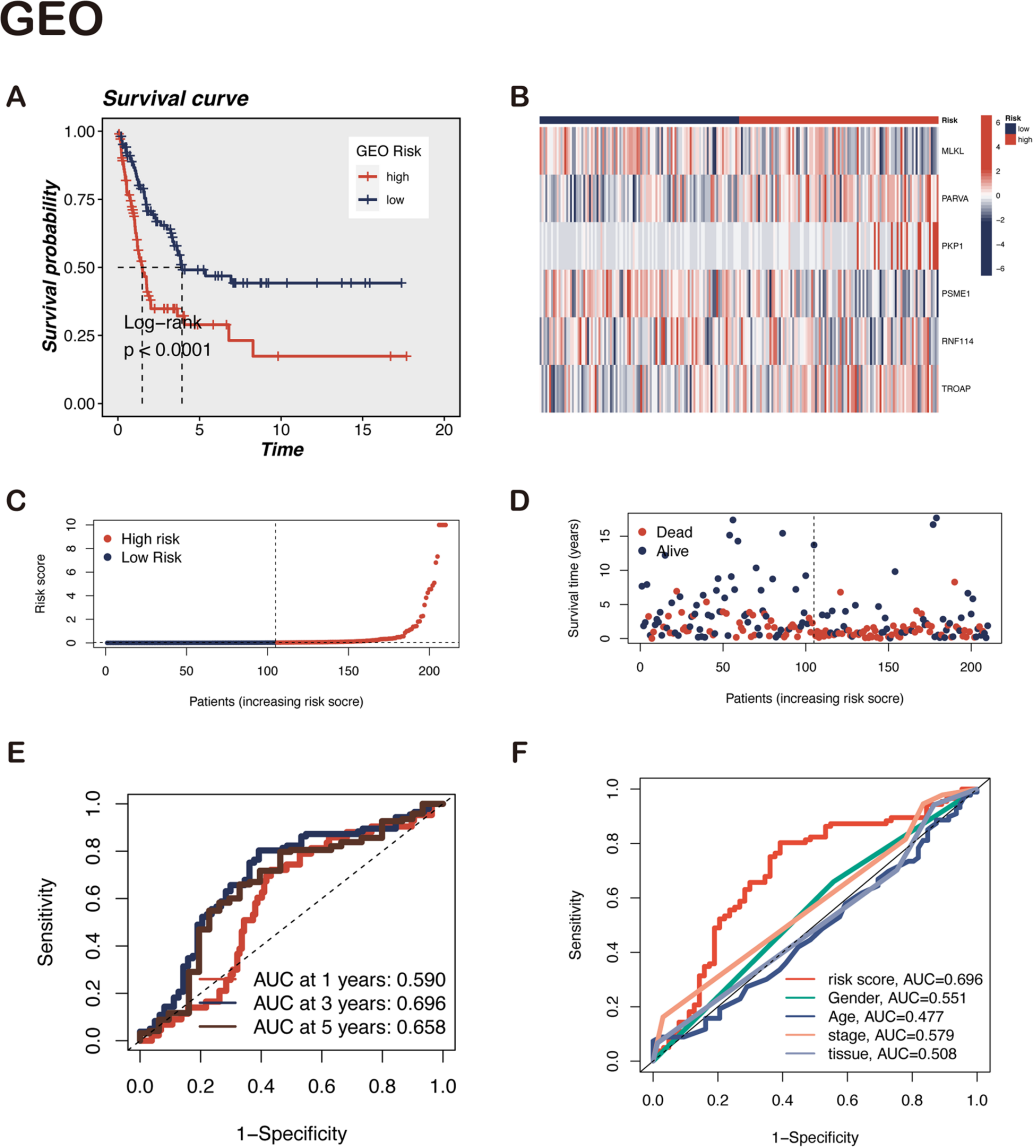
Verification of the accuracy of LLPS-related gene signature using the GEO cohort. **A** Kaplan–Meier curves showed the survival differences between the high-risk and low-risk groups. **B** The expression levels of six candidate genes in the two risk groups. **C**, **D** The distribution of risk score and survival time in the high- and low-risk groups. **E**, **F** The efficacy of the risk score in predicting overall survival through the ROC curve and the AUC of risk score was 0.696 compared to other clinical parameters

To investigate the reliability of the gene signature constructed from the TCGA-SKCM cohort, independent external validation using the GEO database was conducted. Risk scores for melanoma patients in GSE65904 were generated using the same method. The patients were classified into low- and high-risk groups based on the median cutoff value. Consistent with our previous findings, the survival analysis reveals that low-risk patients have a better prognosis than those with high risk (Fig. 4A). The distribution of risk score, survival status, and expression of six hub genes in the GSE65904 set is shown in Fig. 4B–D. ROC curves displayed the 1-year AUC to be 0.590, 3-year AUC 0.698, and 5-year AUC 0.658 (Fig. 4E). Furthermore, compared with multiple clinicopathological factors (gender, age, stage, and tissue), the risk score demonstrates the largest AUC for 3-year OS, suggesting the LLPS-related gene prognostic signature for prognostic prediction of melanoma is highly reliable (Fig. 4F).

### Evaluation of six-gene prognostic signature as an independent prognostic indicator for melanoma patients

**Fig. 5:**
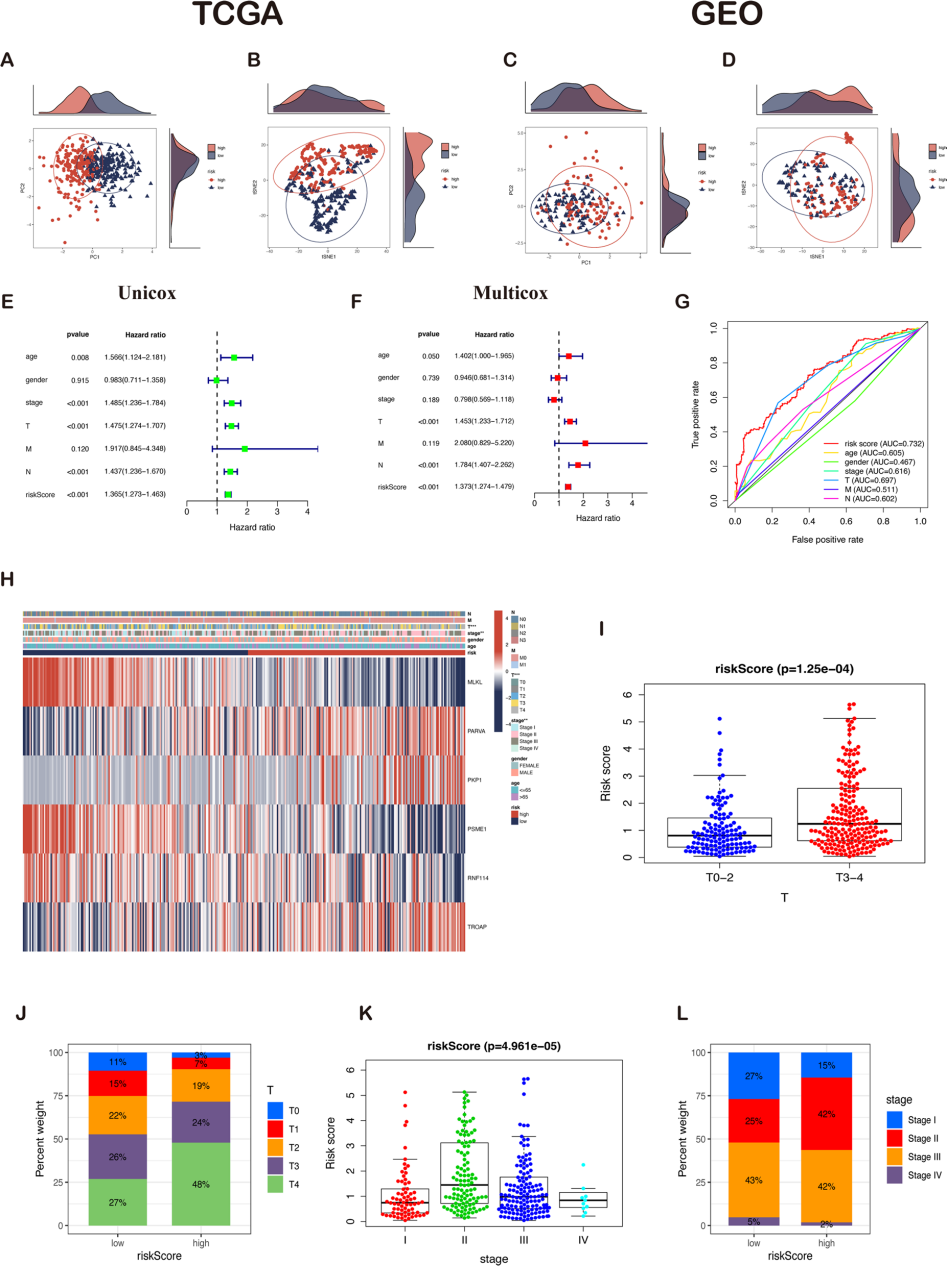
Prognostic value of clinicopathological factors and risk scores in the TCGA cohort. **A** The PCA plot in the TCGA cohort. **B** The t-SNE plot in the TCGA cohort. **C** The PCA plot in the GEO cohort. **D** The t-SNE plot in the GEO cohort. **E** Univariate Cox analysis showed that risk score (*p* < 0.001, HR = 1.365, 95% CI: 1.273–1.463) was associated with OS. **F** Multivariate Cox analysis demonstrated risk score (*p* < 0.001, HR = 1.373, 95% CI: 1.274–1.479) was independently correlated with OS. **G** The ROC curve of the risk score had the largest AUC of 0.732 compared with the other clinical variables. **H** Heatmap (blue: low expression; red: high expression) for the relationship between clinicopathologic features and the risk groups, indicating that T and stage were significantly different between the two groups. **I** Patients with melanoma of T3-4 had significantly higher risk scores than those with T1-2. **J** Patients with T3 and T4 stage accounted for the largest proportion in the high-risk group. **K** Risk scores of patients with different stages had significant differences. **L** Stage III melanoma patients accounted for the largest proportion in the low-risk group, and patients with stage II melanoma in the high-risk group increased significantly compared to the low-risk group. Note: *** *P* ≤ 0.001. ** *P* ≤ 0.01. * *P* ≤ 0.05

First, PCA and t-SNE analyses indicate that six LLPS-related genes have prominent discriminatory power and could better identify low-risk from high-risk populations (Fig. 5A–D). Then, univariate and multivariate Cox regression analyses identified the T classification, pathological N classification, and risk scores as the only three independent prognostic factors (Fig. 5E, F). Moreover, the risk model exhibited superior performance than age, gender, stage, T, M, and N in predicting melanoma prognosis (Fig. 5G). Among these modeling genes, PARVA, PKP1, and TROAP were upregulated, while MLKL, PSME1, and RNF114 were downregulated in the high-risk group (Fig. 5H). Further, the discrepancies in risk scores between subgroups were explored in light of clinicopathological parameters. A significant difference in the distribution of the stage and T classification in the risk group was observed, while other clinical characteristics did not significantly change between the two subgroups. Patients with stage T3–4 melanoma are related to a significantly higher risk score than those with T1–2 classification (Fig. 5I–J). Patients with stage II or III have a higher risk score, indicating our risk model possesses specific clinical significance based on LLPS-related genes (Fig. 5K–L). Therefore, the prognosis of melanoma patients can be determined to a certain extent by this prognostic model.

### Tumor classification based on six-gene signature

**Fig. 6:**
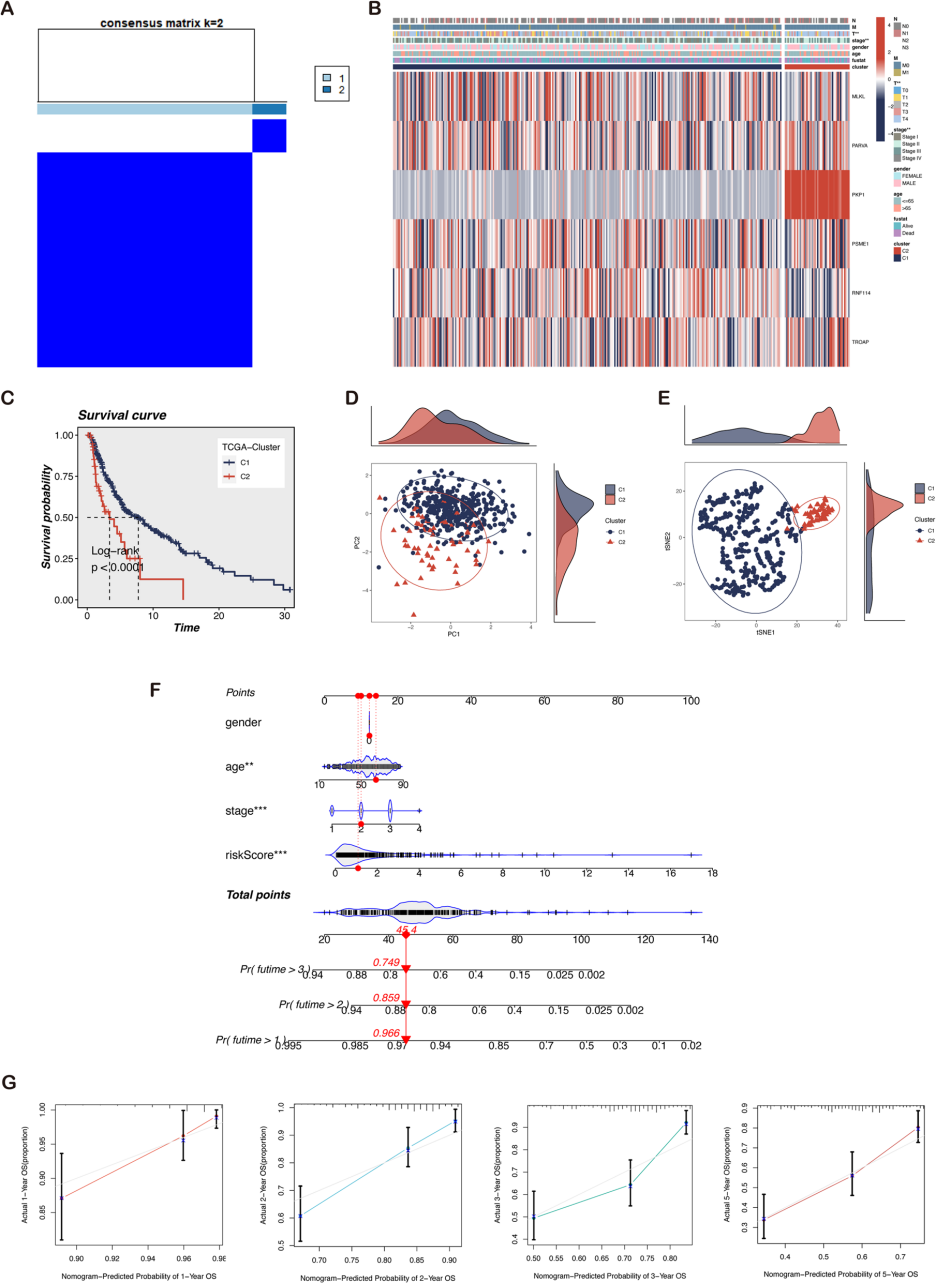
Tumor classification based on the identified prognostic LLPS-associated genes. **A** Stratification of 447 melanoma patients into two clusters according to the consensus clustering matrix (k = 2). **B** Heatmap and the clinicopathologic characteristics of the two clusters. **C** Kaplan–Meier curves showed the OS of cluster 1(blue) and cluster 2 (yellow) for melanoma patients. Cluster 2 had a worse prognosis than cluster 1 (*P* < 0.001). **D** The PCA plot between two clusters. **E** The t-SNE plot between two clusters. **F** Nomogram for the prediction of 1-, 3-, and 5-year survival probability in patients with melanoma. **G** The calibration plots test consistency between the actual OS rates and the predicted survival rates, with the 45°line representing the best prediction. Note: *** *P* ≤ 0.001. ** *P* ≤ 0.01. * *P* ≤ 0.05

The K-means clustering method was applied to analyze a cluster of six modeling genes. The optimal clustering coefficient was determined as two, and then a total of 447 patients were separated into two subtypes (Fig. 6A, the detailed classification process is shown in Fig. S2A). As shown in Fig. 6B, significant differences exist among subtypes regarding stage and T classification. Subsequently, a significant survival difference is indicated between the two categories in Fig. 6C, with cluster 1 having a better prognosis than cluster 2. The Sankey diagram demonstrates that cluster 2 patients predominantly belong to the high-risk group, implying that cluster 2 patients have higher risk scores. This explains the reason behind the poor prognosis of cluster 2 (Fig. S2B). PCA and t-SNE demonstrate that patients with varying clusters are positioned in distinct groups (Fig. 6D, E). A nomogram based on the entire TCGA cohort is established to predict 1-, 3- and 5-year OS. Age, stage, and risk score are the predictors of the nomogram (Fig. 6F). The higher the total points, the worse the clinical outcome for the patients. Figure 6G indicates that the survival times estimated by the nomogram are highly congruent with the predicted survival times. In conclusion, the risk score was an independent predictive indicator and predicted the survival probability of melanoma patients.

### Multi-omics analysis of LLPS-related prognostic genes in melanoma

**Fig. 7:**
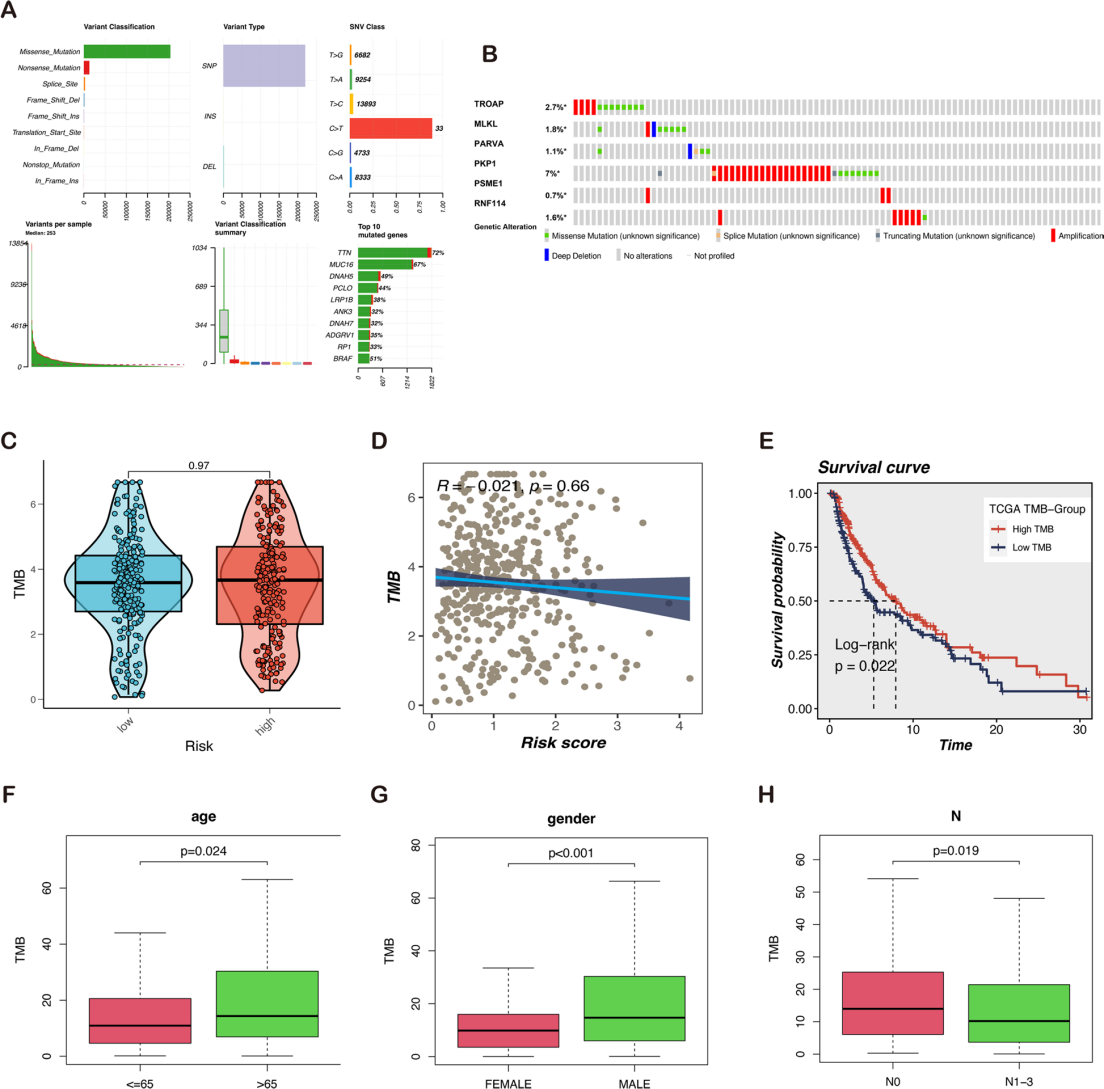
The landscape of melanoma sample mutation profiles. **A** Description of the statistical measurement mutation details, among which the most common mutation type was a missense mutation. SNP occupied an absolute proportion compared with insertion or deletion, and C > T occurred more frequently than in other classifications of forms. The horizontal histogram listed the top 10 mutated genes in melanoma. **B** Genetic alterations of the six LLPS-related genes in melanoma were analyzed by cBioPortal. **C** Comparison of tumor mutation burden (TMB) between different risk groups. **D** Correlation analysis between risk score and TMB. **E-H** TMB and association with clinical features. Low levels of TMB correlated with poorer survival with *p* = 0.022. High TMB levels were associated with age, gender, and pathological N classification

The overview of mutations in the SKCM samples is shown in Fig. 7A. Among the mutations shown, the most common type is a missense mutation. Single nucleotide polymorphism (SNP) occupies an absolute proportion compared with insertion or deletion, and C > T occurs more frequently than in other categories. Therefore, the median number of mutation variants per sample was 253, and box plots of each color represent various mutation types. In addition, the horizontal histogram displayed the top 10 mutated genes, including TNN (72%), MUC16 (67%), BRAF (51%), and DNAH5 (49%). Furthermore, using cBioPortal, the mutation rates of six signature genes are found to be low (Fig. 7B). Specifically, PKP1 ranked as the most frequently altered gene. PKP1 and TROAP were commonly amplified in melanoma patients. It is to be noted that high TMB levels contribute to tumors expressing recognized neo-antigens and augmenting immune responses [26]. The TMB value neither significantly differs between the two risk groups, nor does any association between risk scores and TMB value exists (Fig. 7C, D). Survival analysis suggests that higher TMB levels are associated with better OS (Fig. 7E) and are positively correlated with older individuals, males, and lower pathological N classification (Fig. 7F–H). However, there is no significant association between TMB and pathological T, M, and stage (Fig. S3A). These findings indicated that melanoma outcomes are improved by a higher TMB level.

### Estimation of tumor immune cell infiltration and immunotherapy response according to the signature

**Fig. 8:**
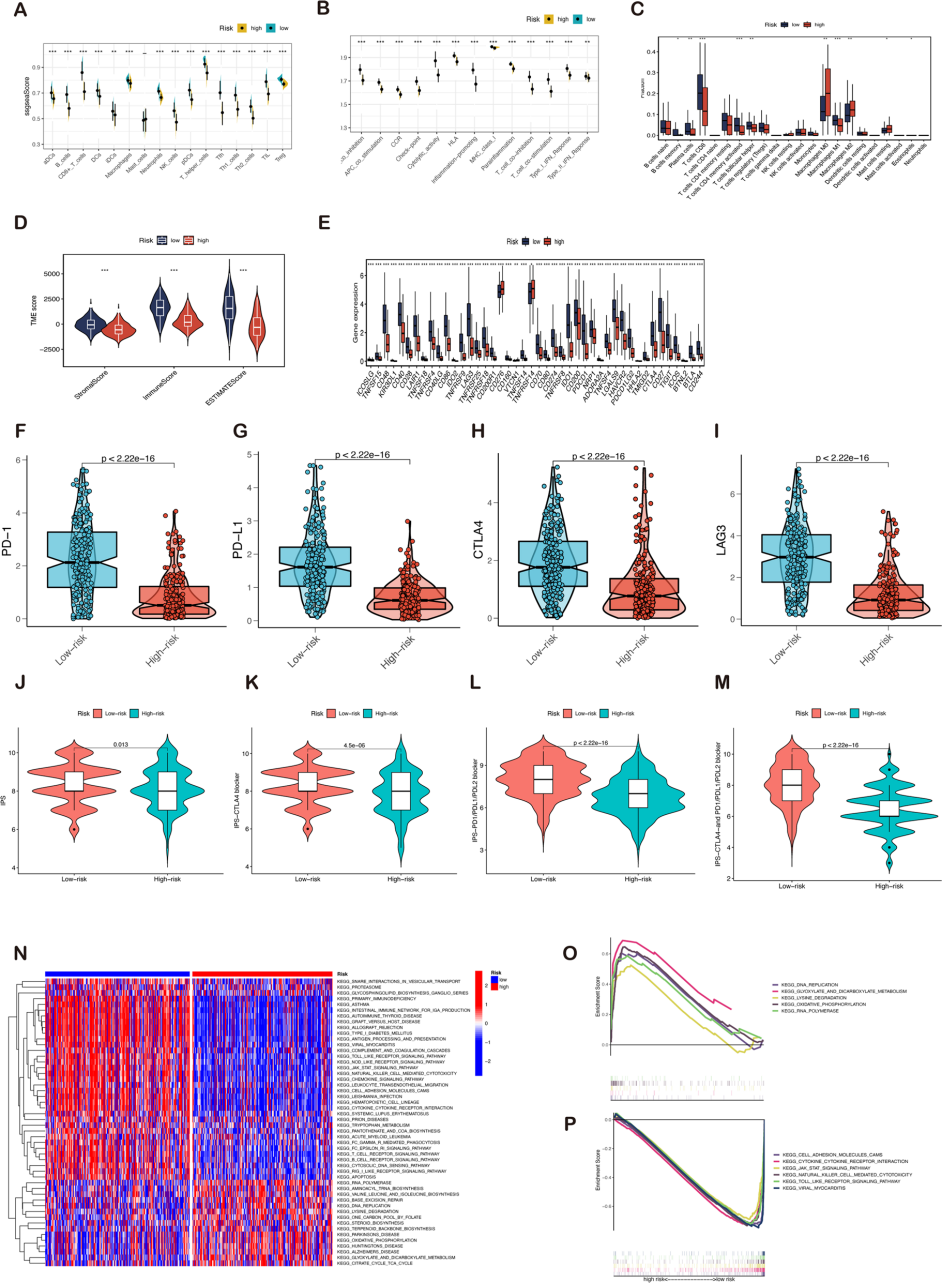
Analysis of immune infiltration, tumor microenvironment, and immunotherapy evaluation. The association between risk score and 16 types of immune cells **A** and 13 immune-related functions **B** in the low (blue box) and high-risk (red box) groups. **C** The proportional differences of specific 22 immune fractions were calculated by the CIBERSORT method in two groups. **D** The violin plot demonstrated the difference in ESTIMATE Score, Immune Score, and Stromal Score calculated using the ESTIMATE algorithm between the two groups. Scores of components in the tumor microenvironment in the low-risk group were significantly higher than those in the high-risk group. **E** Association of 47 immune checkpoint gene expression with risk score levels in melanoma. The mRNA expression of **F** PD-1, **G** PD-L1, **H** CTLA4, and **I** LAG3 in the low-risk group were significantly higher than that in the high-risk group. The low-risk subtype has significantly greater IPS **J**, IPS-CTLA4 blocker **K**, IPS-PD1/PDL1/PDL2 blocker **L**, and IPS-CTLA and PD1/PDL1/PDL2 blocker **M** compared to the high-risk subtype. **N** The overview of gene set enrichment analysis between risk groups. **O** Metabolism-related pathways were concentrated in the high-risk group. **P** Immune regulation and tumor-associated signaling pathways were enriched in the low-risk group. Note: *** *P* ≤ 0.001. ** *P* ≤ 0.01. * *P* ≤ 0.05

To further explore the associations between the immune status and melanoma risk score, the infiltration scales of diverse immune cells were qualified with ssGSEA according to the specific reference gene sets. Figure 8A and B demonstrate that the degree of almost all immune cell infiltration and immune function is substantially higher in patients with low-risk scores. Since the variations in immune infiltration contribute to identifying differences in the intrinsic characteristics of individuals, the relationship between the melanoma samples and immune infiltration was measured (Fig. S3B). The CIBERSORT algorithm reveals that the high-risk subgroup patients exhibit a higher proportion of M0 and M2 macrophages and resting mast cells as shown in Fig. 8C. In contrast, plasma cells, CD8^+^T cells, activated CD4^+^T cells, Th cells, and Macrophages M1 are predominantly infiltrated in the low-risk group. The ESTIMATE algorithm corroborates the above results, demonstrating that the low-risk group has comparatively higher immune scores, stromal scores, and estimate scores (Fig. 8D). The score of each component in the tumor microenvironment is strongly and adversely correlated with risk scores. Furthermore, the immune checkpoint-associated gene expression is more active in the low-risk group (Fig. 8E). From these findings, it can be concluded that samples with diverse risk scores exhibit remarkably different immunological properties. Typically, low-risk patients exhibit an immune-excluded condition (stromal activation and abundant immune infiltration). In contrast, samples with a high-risk score were associated with an immunological-desert environment, distinguished by decreased immune infiltration.

The most effective anticancer therapies for melanoma currently being investigated are ICIs [27]. The difference in PD-1, PD-L1, CTLA4, and LAG3 among different groups was further explored to provide a deeper understanding of immunotherapy. The expressions of PD-1, PD-L1, CTLA4, and LAG3 were found to be significantly higher in patients with low-risk scores (Fig. 8F–I). Furthermore, the IPS can contribute to screening patients who are susceptible to immunotherapy. In the current study, the low-risk subtype has significantly higher IPS and other blocker scores compared with the high-risk subtype. This highlights that low-risk patients may be more sensitive to ICIs treatment and derive comparatively greater benefits than those belonging to the high-risk group (Fig. 8J–M).

To identify the possible pathways connected with the risk score in melanoma, GSVA was conducted to explore the enriched KEGG pathways using the risk level as phenotype. As shown in Fig. 8N, “DNA replication”, “RNA polymerism”, “Oxidative-phosphorylation”, and “Glyoxylate and dicarboxylate metabolism” are significantly associated with the high-risk group. In contrast, the low-risk group mainly focuses on the “JAK-STAT signaling pathway”, the “cell adhesion molecules cams”, “Toll-like receptor signaling pathway”, and “NK cell-mediated cytotoxicity”. We also chose partial top GSEA results shown in Fig. 8O–P.

### Diagnosis, prognosis, and immune infiltration of TROAP in melanoma

**Fig. 9:**
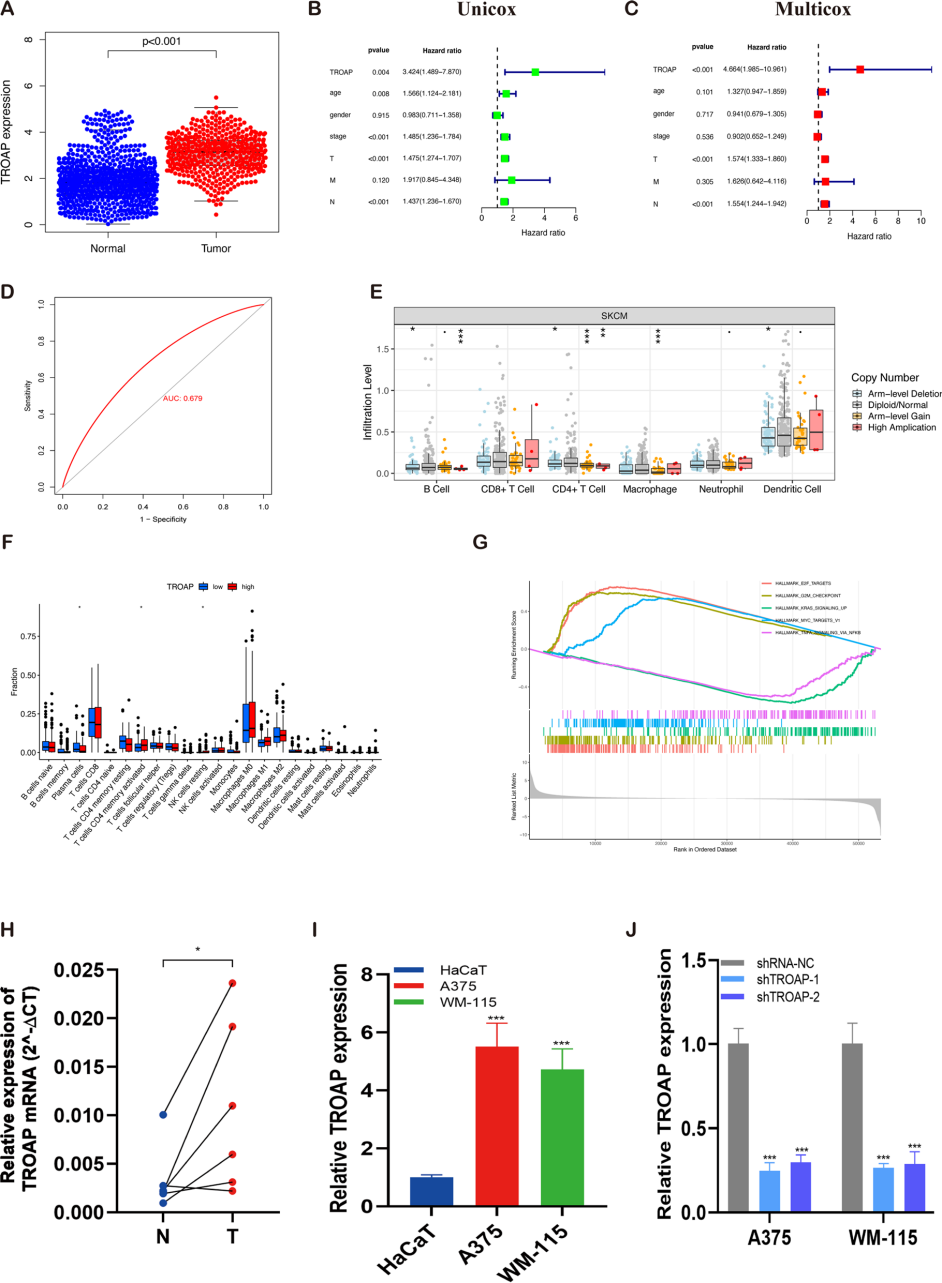
TROAP expression and prognostic value in melanoma. **A** TROAP was highly expressed in melanoma samples compared to the corresponding normal tissues of the GTEx-skin dataset as a control. The univariate **B** and multivariate **C** Cox regression analysis revealed the role of TROAP in predicting melanoma survival. **D** TROAP has a robust diagnostic accuracy for melanoma with an AUC value of 0.679 for the ROC curve. **E** TROAP copy number variation (CNV) correlates with immune cell infiltration levels in the TIMER database. **F** The violin represented immune cell infiltration in the low TROAP expression (blue) and the high TROAP expression (red) groups. **G** GSEA enrichment plots represented enriched biological pathways in high and low TROAP groups. **H** RT-qPCR analysis of TROAP expression of mRNA in 6 paired fresh melanoma tissues (T) and matched adjacent normal tissues (N) quantifed after transfection. **I** Compared with HaCaT, TROAP was up-regulated in A375 and WM-115 melanoma cell lines. **J** RT-qPCR was performed to measure the relative expression of TROAP in transfected with sh-RNAs or negative control (NC). Note: *** *P* ≤ 0.001. ** *P* ≤ 0.01. * *P* ≤ 0.05

Melanoma samples from the TCGA and GTEX integrated datasets demonstrated a high level of TROAP expression (Fig. 9A). The TROAP and OS were linked in the univariate analysis (HR = 3.424, 95% CI = 1.489–7.870, *p* = 0.04, Fig. 9B) and multivariate Cox regression analysis (HR = 4.664, 95% CI = 1.958–10.961, *p* < 0.001, Fig. 9C). Moreover, patients with high TROAP expression had a considerably worse prognosis than those with low TROAP expression. A ROC curve was utilized to evaluate the accuracy of the TCGA database in diagnosing melanoma. An AUC value of 0.679 suggests that TROAP has an excellent diagnostic value for melanoma (Fig. 9D). The diverse forms of copy number variation (CNV) carried by TROAP can generally inhabit immune infiltrates, including B cells, CD4 + T cells, macrophages, and dendritic cells (Fig. 9E). The relationship between the TROAP expression and abundance of the infiltrated immune cells was explored using the CIBERSORT algorithm. Activated CD4 + T cells, plasma cells, and resting NK cells are more abundant in the high TROAP expression group (Fig. 9F). Finally, GSEA indicated that hallmark pathways are most involved with E2F targets, G2M checkpoint, and MYC targets V1 in the high-TROAP groups, while KRAS signaling up and TNFA signaling via NF-kB are significant in low-TROAP groups (Fig. 9G).

### Inhibition of proliferation, invasion, and migration of melanoma cells by TROAP knockdown

**Fig. 10:**
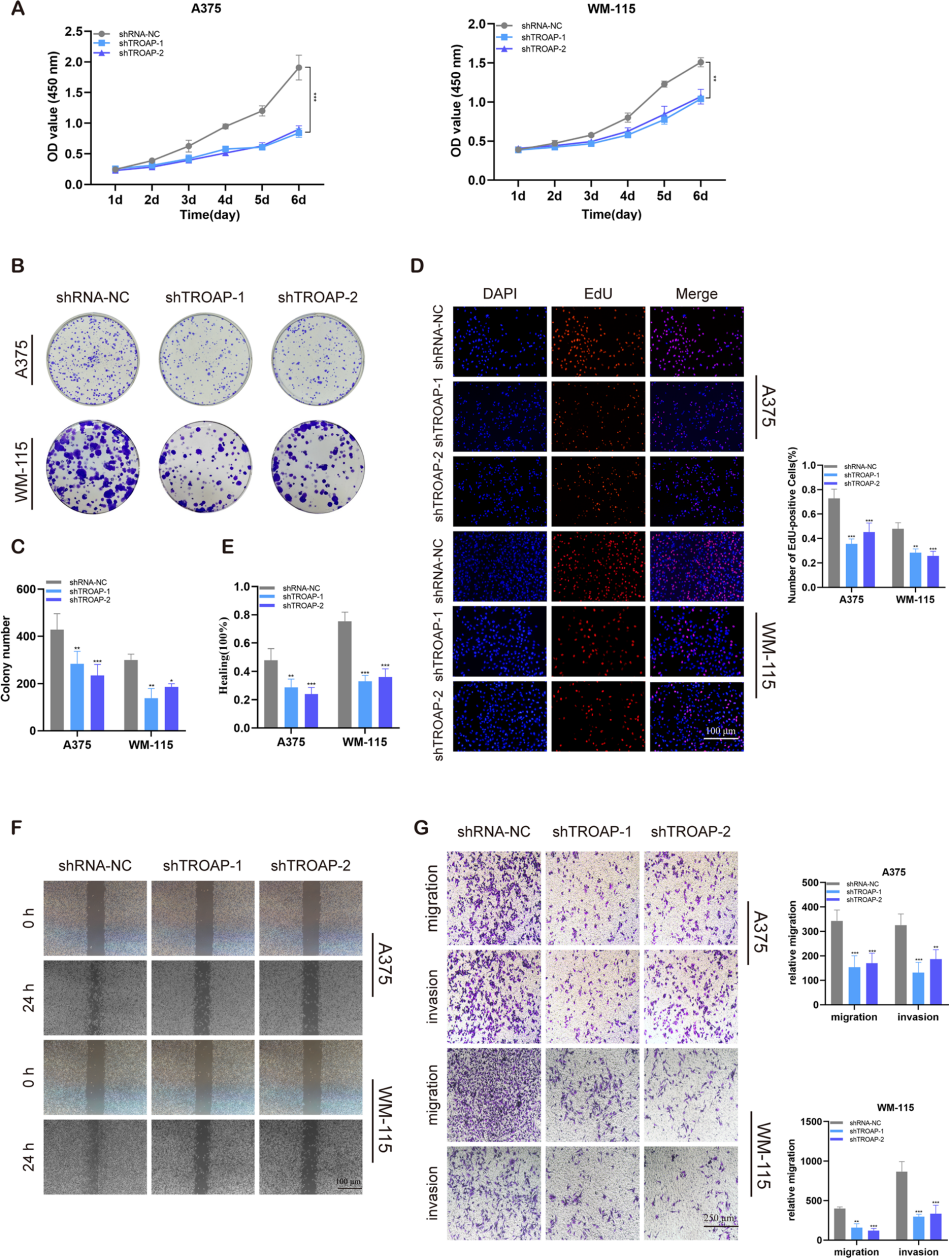
Effect of TROAP knockdown on melanoma cell proliferation and migration in vitro. **A** CCK-8 assays showed that TROAP inhibition reduced melanoma cells (A375 cell line and WM-115 cell line) proliferative ability in vitro. **B**, **C** Colony formation assay displayed cells with reduced TROAP expression exhibited a significant reduction in the numbers of colonies compared with the shRNA NC group. **D** EdU staining assay indicated that downregulation of TROAP expression repressed A375 cells and WM-115 cells proliferation. (Scale bar, 100 μm). **E**, **F** Scratch-wound healing assay indicated a significantly slower wound healing rate in cells with a reduced expression of TROAP (Scale bar, 100 μm). **G** Transwell assay showed that downregulation of TROAP expression inhibited the migration and invasion capacity of A375 cells and WM-115 cells (Scale bar, 250 μm). Note: *** *P* ≤ 0.001. ** *P* ≤ 0.01. * *P* ≤ 0.05

The validation of mRNA expression levels of TROAP was performed, which indicated a significant increase in TROAP expression in our clinical melanoma specimens compared with paired normal tissues (Fig. 9H). This finding was consistent with the results from previous database analysis. Meanwhile, the same tendency of TROAP expression is observed in melanoma cell lines and normal skin cell HaCaT (Fig. 9I). Furthermore, we observed weakly positive or negative TROAP in the cytoplasm of most malignancies. Moderate to strong membranous and cytoplasmic staining was observed in ovarian cancers, colorectal, prostate, and liver cancers according to the HPA database (Table S4). We selected two cases of TROAP in melanoma with IHC pictures and found TROAP was positively expressed in skin melanocytes and melanoma (Figure S4). The TROAP lentiviral vector in A375 and WM-115 cells was transfected, and the transfection efficiency of TROAP was detected by qRT-PCR. The expression of TROAP was dramatically decreased in A375 and WM-115 cells after sh-TROAP tranfection (Fig. 9J). The proliferation ability of TROAP on melanoma cells was evaluated by CCK-8 and EdU assay, the results from which indicate that compared to the control group, the viability and EdU positive cell ratio declined in A375 and WM-115 cells transfected with sh-TROAP (Fig. 10A, D). This implied that TROAP may exert a potentially pivotal influence on the proliferation of melanoma cell lines. Similarly, the colony formation test demonstrates that TROAP downregulation exhibits a remarkable reduction in the number of colonies relative to the control group (Fig. 10B, C). Next, the wound healing assay yields a similar result that indicates the wound healing rate is markedly decreased by silencing TROAP (Fig. 10E, F). The findings from Transwell invasion and migration experiments indicate that TROAP knockdown decreases the percentage of cells migrating through the transwell plate. Moreover, TROAP downregulation dramatically inhibits the ability of A375 and WM-115 cells to invade and migrate (Fig. 10G). In conclusion, TROAP was found to play a significant role in melanoma proliferation, invasion, and metastasis, thus supporting the findings of the bioinformatics study.

### TROAP promotes tumor growth of A375 cells in vivo

**Fig. 11:**
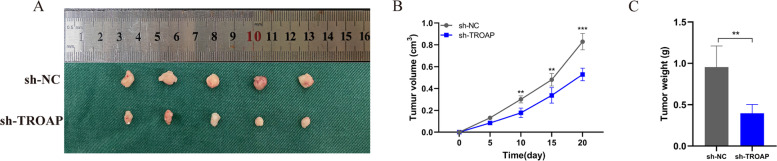
Low expression of TROAP inhibits melanoma growth in vivo. **A** Photographs of tumors obtained from the different groups of nude mice transfected with sh-NC and sh-TROAP. The average weight and tumor size were used to observe tumors. **B** The growth curves were determined by measuring tumor volumes every five days. **C** Knockdown of TROAP expression significantly inhibited melanoma cancer cell growth in nude mice and tumor weight was significantly reduced in the sh-TROAP group compared to that in the NC group. Note: *** *P* ≤ 0.001. ** *P* ≤ 0.01. * *P* ≤ 0.05

As above-mentioned, the model of knockdown of TROAP could be successfully built following lentivirus transfection. In order to investigate the effects of TROAP expression on tumor growth in vivo, A375 cells transfected with TROAP or negative control were injected subcutaneously into nude mice xenograft model respectively. After 20 days of tumor implantation, TROAP silencing significantly reduced tumor growth (Fig. 11A). The tumor weight and volume were also significantly reduced due to TROAP silencing (Fig. 11B, C). These findings suggest that TROAP silencing in vivo significantly impaired melanoma tumorigenicity.

## Discussion

In 2012, Pilong Li discovered that RNA and protein molecules might undergo droplet-like fusion through weak interaction forces, demonstrating that biochemical interactions can generate phase separation in vitro [17]. Evidence suggests that altered phase separation is intricately linked to cancer formation and progression. To date, only a single study has directly investigated the relationship between protein phase separation and cancer [28]. Herein, cancer mutations in the tumor suppressor speckle-type BTB/POZ protein (SPOP) were found to be associated with specific phase separation defects. SPOP is a substrate adaptor for cullin 3 RING ubiquitin ligase (CRL3) that targets a variety of proto-oncoproteins for ubiquitination and proteasome degradation. Cancer mutations disrupt SPOP-substrate interaction, thereby disrupting SPOP phase separation and localization of membrane-free organelles [29]. Several malignancies occur because of chromosomal translocations leading to new gene products, such as fusion proteins. The phase separation provides a mechanism for the fusion proteins to drive abnormal gene expression programs [28, 30]. For instance, the LLPS of intrinsically disordered regions (IDRs) in NUP98-HOXA9 promotes the activation of oncogenes that induces mutations and carcinogenesis [30]. LLPS opens new avenues for the understanding of cancer phenotypes and has the potential to become a new tumor therapeutic target [31].

Scholars are increasingly concerned about the potential prognostic value of LLPS-related genes. In this study, the prognostic characteristics of LLPS-related genes in melanoma were constructed through an extensive analysis of melanoma data from the TCGA and GEO databases. The patients with high-risk scores in TCGA and GEO cohorts were found to exhibit worse outcomes. Moreover, the risk score is observed to be strongly positively linked with the clinical stage and T classification. In our perception, the later the stage, the worse the prognosis, which confirms our findings that patients with high-risk scores have a poor prognosis. Therefore, the immune landscapes of high- and low-risk populations were compared using the CIBEROST, ESTIMATE, and ssGSEA algorithms to determine potential causes of the prognosis variance. The results demonstrated that nearly all immune cell infiltration, immune scores, and immune-relevant characteristics were dramatically active in patients with low-risk scores. Meanwhile, GSEA revealed that the functional pathways involved in the low-risk group were mainly immune regulation and tumor-associated signaling pathways. Therefore, the tumor immunosuppressive microenvironment probably generates adverse outcomes in high-risk patients.

Immunotherapy has dramatically improved cancer treatment and revitalized the field of tumor immunology [32]. ICIs with PD1, PD-L1, CTLA4, and LAG3 are currently considered important targets in medical practice and have become vital immunotherapy [33, 34]. ICIs maintain immune tolerance and evade immune surveillance by inhibiting molecules of signaling pathways. Patients with melanoma sensitive to ICIs therapy have better prognoses. However, not all patients have a lasting response to ICIs treatment. Therefore, the identification of eligible patients is urgently needed. The current study validated that critical targets of ICIs therapy were highly overexpressed in patients with low-risk scores. Similarly, the low-risk group exhibited a higher IPS than the high-risk group, implying higher tumor immunogenicity and strong immune response in the low-risk scores populations. These findings implicated that immunotherapy may be more effective in the low-risk group than the high-risk one. Hence, the LLPS-related gene signature could be used to identify melanoma patients who would benefit from ICIs therapy.

Because of the complexity of the immune system, the combination of multiple biomarkers is required to predict ICIs outcomes and fully unlock the potential benefit of immunotherapy. Numerous studies have demonstrated that elevated TMB levels in tumors are associated with an additional neo-antigen formation that renders tumors more immunogenic, enhancing clinical response to immunotherapy [35–37]. Thus, TMB has been a novel biomarker of immunotherapeutic responsiveness in melanoma [38]. Our research observed a correlation between elevated TMB levels and improved clinical outcomes. Besides, higher TMB levels were associated with higher age, male, and lower-N classification. We considered that patients with higher TMB levels could benefit more from immunotherapy than those with lower TMB levels.

Further, the percentage of each stage was quantified in the low-risk group and it was found that stage III melanoma patients formed the most significant proportion. Adjuvant therapy based on ICIs and targeted agents significantly improves patients with stage III melanoma [33], consistent with our previous speculation that immunotherapy was more suitable for the low-risk group. In addition, in comparison to the low-risk groups, the proportion of stage II was found to be much higher in the high-risk groups. Therefore, the focus of treatment for high-risk groups should shift to stage II melanoma. GSEA indicated that metabolism-related pathways were predominantly enriched in the high-risk population. Simultaneously, based on the data of the large phase III clinical trial KEYNOTE-716 [39], pembrolizumab was included in the 2022 Melanoma Treatment Guidelines for the adjuvant treatment of resected high-risk stage II Melanoma. Hence, early measures should be adopted for patients with stage II melanoma, and those combined with metabolic interventions to disrupt the metabolism of high-risk groups may improve the efficacy of ICIs therapy in melanoma. However, these results require further clinical validation in a larger cohort and different centers.

Through LASSO regression, the following six genes were specifically identified to have an irreplaceable prognostic effect on melanoma patients: MLKL, PARVA, PKP1, PSME1, RNF114, and TROAP. It is to be noted that functional studies of these six genes in melanoma are limited. Therefore, our discovery of the candidate key genes of melanoma is significant from the perspective of LLPS. The associations of these genes to other cancers may enable us to study their functions in melanoma. TROAP with the highest HR value was selected as the validation gene. TROAP, a cytoplasmic protein consisting of 778 amino acid residues, is essential for maintaining the structural and dynamic features of centrosomes, thereby contributing to spindle bipolarity during mitosis [40, 41]. Previous studies reported high TROAP mRNA levels in the bone marrow, skin, esophagus, spleen, testis, and 11 other tissues (http://www.ncbi.nlm.nih.gov/gene/10024). Consistent with these findings, high TROAP expression enhances malignancy and is involved in the poor prognosis of glioma [42], liver cancer [43], lung cancer [44], prostate cancer [45], and gastric cancer [46]. Nevertheless, only a few researchers have focused on the prognostic role of TROAP in melanoma. The current research identified it as an LLPS-related gene and investigated it through experiments. TROAP severed as a cycling protein essential for mitosis and its endogenous expression was carefully regulated during cell cycle development. Herein, the E2F target and G2M checkpoint pathways were found to belong to the active signaling pathways when TROAP was highly expressed. The TROAP expression in melanoma cells was dramatically higher than that in normal cells. A range of experiments studying cell functions indicated that the ability of melanoma cells to grow, proliferate, invade and migrate were declined by knocking down TROAP. Therefore, TROAP can become a potential target in melanoma treatment.

Although a predictive model was built based on six LLPS-related genes in melanoma patients, the current work has several limitations. First, the LLPS-related genes were proposed based on a retrospective study. Hence, prospective studies are necessary to verify before proceeding to clinical decision-making. Second, only the expression and biological function of TROAP were validated herein, the mechanism of how TROAP influences melanoma cell proliferation and migration remains to be understood via future research. Further clinical investigations are imperative to elucidate the molecular mechanisms underlying the role of TROAP in melanoma progression.

## Conclusions

A comprehensive bioinformatic analysis was performed and a predictive model for melanoma risk stratification was identified based on six LLPS-related genes. The prognosis and immunological microenvironment of melanoma patients were assessed via this model, and it was speculated that LLPS was a promising biomarker for predicting the efficacy of melanoma immunotherapy. The role of TROAP in melanoma was also confirmed by cell and animal assays, which may provide useful insight into melanoma treatment strategies.

## Supplementary Information

**Additional file 1.**

## Data Availability

The datasets used and/or analysed during the current study are available from the corresponding author on reasonable request.

## Abbreviations

LLPS: Liquid–liquid phase separation
TCGA: The Cancer Genome Atlas
ICIs: Immune checkpoint inhibitors
SKCM: Skin cutaneous melanoma
GTEX: Genotype-Tissue Expression
GEO: Gene Expression Omnibus database
MLOs: Membrane-less organelles
OS: Overall survival
LASSO: Least absolute shrinkage and selection operator
t-SNE: T-distributed stochastic neighbor embedding
PCA: Principal component analysis
AUC: Area under the curve
ROC: Receiver operating characteristic
TMB: Tumor mutation burden
ssGSEA: Single-sample gene set enrichment analysis
TICs: Tumor-infiltrating immune cells
TME: Tumor microenvironment
IPS: Immunophenoscore
TCIA: The Cancer Immunome Atlas
GSEA: Gene set enrichment analysis
KEGG: Kyoto Encyclopedia of Genes and Genomes
HaCaT: Human keratinocyte cells
FBS: Fetal bovine serum
RT-PCR: RNA extraction and quantitative real-time PCR
DEPC: Diethylpyrocarbonate
CCK-8: Cell counting kit-8
PBS: Phosphate buffer saline
SD: Standard deviation
PD-1: Programmed cell death protein 1
PD-L1: Programmed cell death-Ligand 1
CTLA4: Cytotoxic T lymphocyte-associated protein 4
LAG3: Lymphocyte-activation-gene-3
GSVA: Gene set variation analysis
CNV: Copy number variation
SPOP: Speckle-type BTB/POZ protein
CRL3: Cullin 3 RING ubiquitin ligase
HR: Hazard ratio
IDRs: Intrinsically disordered regions
