# Supplementary material for: Liquid-liquid phase separation throws novel insights into treatment strategies for skin cutaneous melanoma
Source: BMC Cancer. 2023 May 1;23:388. doi: 10.1186/s12885-023-10847-w (PMC10150491; doi:10.1186/s12885-023-10847-w)
Supplement: Supplementary file 1 — Additional file 1. [file 12885_2023_10847_MOESM1_ESM.zip › Supplementary file/Table S4.docx]

**Table S4 Immunohistochemical analysis of TROAP in The Human Protein Atlas database**

| Gene | Gene.name | Cancer | High | Medium | Low | Not.detected |
| --- | --- | --- | --- | --- | --- | --- |
| ENSG00000135451 | TROAP | breast cancer | 0 | 0 | 2 | 9 |
| ENSG00000135451 | TROAP | carcinoid | 0 | 0 | 0 | 4 |
| ENSG00000135451 | TROAP | cervical cancer | 0 | 4 | 6 | 2 |
| ENSG00000135451 | TROAP | colorectal cancer | 0 | 5 | 4 | 2 |
| ENSG00000135451 | TROAP | endometrial cancer | 1 | 1 | 6 | 3 |
| ENSG00000135451 | TROAP | glioma | 0 | 0 | 0 | 11 |
| ENSG00000135451 | TROAP | head and neck cancer | 0 | 0 | 1 | 3 |
| ENSG00000135451 | TROAP | liver cancer | 0 | 4 | 3 | 5 |
| ENSG00000135451 | TROAP | lung cancer | 0 | 2 | 3 | 6 |
| ENSG00000135451 | TROAP | lymphoma | 0 | 0 | 0 | 12 |
| ENSG00000135451 | TROAP | melanoma | 0 | 0 | 2 | 10 |
| ENSG00000135451 | TROAP | ovarian cancer | 4 | 5 | 3 | 0 |
| ENSG00000135451 | TROAP | pancreatic cancer | 0 | 5 | 3 | 3 |
| ENSG00000135451 | TROAP | prostate cancer | 0 | 3 | 2 | 7 |
| ENSG00000135451 | TROAP | renal cancer | 0 | 0 | 1 | 11 |
| ENSG00000135451 | TROAP | skin cancer | 0 | 0 | 2 | 9 |
| ENSG00000135451 | TROAP | stomach cancer | 0 | 0 | 2 | 9 |
| ENSG00000135451 | TROAP | testis cancer | 0 | 2 | 8 | 0 |
| ENSG00000135451 | TROAP | thyroid cancer | 0 | 0 | 2 | 2 |
| ENSG00000135451 | TROAP | urothelial cancer | 0 | 6 | 2 | 3 |
