# Supplementary material for: Liquid-liquid phase separation throws novel insights into treatment strategies for skin cutaneous melanoma
Source: BMC Cancer. 2023 May 1;23:388. doi: 10.1186/s12885-023-10847-w (PMC10150491; doi:10.1186/s12885-023-10847-w)
Supplement: Supplementary file 1 — Additional file 1. [file 12885_2023_10847_MOESM1_ESM.zip › Supplementary file/Table S3.docx]

**Table S3A. Genes related to OS were screened out by univariate Cox regression**

| **Gene** | **HR** | **HR.95L** | **HR.95H** | ***p*-value** |
| --- | --- | --- | --- | --- |
| A2M | 0.831229 | 0.751709 | 0.919162 | 0.000315 |
| ACAP1 | 0.711026 | 0.603862 | 0.837208 | 4.28E-05 |
| ACSL4 | 0.679816 | 0.57695 | 0.801022 | 4.02E-06 |
| ACTR3 | 0.658272 | 0.518716 | 0.835375 | 0.000583 |
| AGAP2 | 0.625377 | 0.474177 | 0.824789 | 0.000887 |
| AHI1 | 0.605867 | 0.464117 | 0.790909 | 0.000229 |
| AKR1B10 | 1.278265 | 1.108601 | 1.473895 | 0.000728 |
| ALG13 | 0.578821 | 0.429131 | 0.780725 | 0.000342 |
| ANXA7 | 0.649248 | 0.508741 | 0.828562 | 0.000518 |
| APOBEC3F | 0.625145 | 0.510397 | 0.76569 | 5.62E-06 |
| APOBEC3G | 0.659878 | 0.572732 | 0.760285 | 8.80E-09 |
| ARG1 | 1.323722 | 1.131001 | 1.549281 | 0.000477 |
| ARRB2 | 0.637504 | 0.504168 | 0.806103 | 0.00017 |
| ATG3 | 0.556983 | 0.42195 | 0.73523 | 3.61E-05 |
| ATRX | 0.642222 | 0.502958 | 0.820046 | 0.000384 |
| BRAF | 0.575096 | 0.424669 | 0.778806 | 0.000349 |
| BTF3L4 | 0.599358 | 0.444974 | 0.807304 | 0.000756 |
| C2orf42 | 0.470193 | 0.30776 | 0.718357 | 0.000484 |
| CALML5 | 1.160209 | 1.088254 | 1.236922 | 5.39E-06 |
| CASP3 | 0.660755 | 0.526433 | 0.82935 | 0.000352 |
| CCNF | 1.496283 | 1.191576 | 1.87891 | 0.000523 |
| CDK2 | 1.174523 | 1.068454 | 1.291122 | 0.000865 |
| CEP290 | 0.63261 | 0.483453 | 0.827786 | 0.000845 |
| CETN3 | 0.614589 | 0.466616 | 0.809487 | 0.000532 |
| CLEC2D | 0.639799 | 0.5145 | 0.795613 | 5.92E-05 |
| CLIC2 | 0.719346 | 0.628635 | 0.823147 | 1.67E-06 |
| CLIC4 | 0.727571 | 0.626657 | 0.844735 | 2.98E-05 |
| CNBP | 0.56917 | 0.436972 | 0.741362 | 2.93E-05 |
| CNOT6L | 0.589154 | 0.448049 | 0.774698 | 0.000152 |
| CNOT8 | 0.615912 | 0.480955 | 0.788739 | 0.000123 |
| COPB1 | 0.651257 | 0.522577 | 0.811623 | 0.000134 |
| COPS2 | 0.693262 | 0.564827 | 0.850903 | 0.000458 |
| COPS5 | 0.623678 | 0.471434 | 0.825087 | 0.000945 |
| CPEB3 | 0.294017 | 0.167412 | 0.516367 | 2.04E-05 |
| CPEB4 | 0.643415 | 0.518264 | 0.798788 | 6.45E-05 |
| CS | 1.761187 | 1.323179 | 2.344188 | 0.000105 |
| CSF1R | 0.779488 | 0.698412 | 0.869977 | 8.76E-06 |
| CSTB | 1.269255 | 1.103575 | 1.459809 | 0.000835 |
| CYLD | 0.569488 | 0.453145 | 0.715702 | 1.37E-06 |
| DDX5 | 0.635563 | 0.50157 | 0.805352 | 0.000175 |
| DDX58 | 0.707454 | 0.596762 | 0.838679 | 6.71E-05 |
| DHX58 | 0.731136 | 0.613713 | 0.871027 | 0.000455 |
| DLG3 | 0.644749 | 0.510958 | 0.813571 | 0.000217 |
| DSC1 | 1.276428 | 1.115583 | 1.460463 | 0.000383 |
| DSG1 | 1.222557 | 1.111483 | 1.34473 | 3.55E-05 |
| DTX3L | 0.690368 | 0.596231 | 0.799367 | 7.27E-07 |
| EAF2 | 0.585824 | 0.473341 | 0.725038 | 8.84E-07 |
| EIF1B | 0.596351 | 0.448754 | 0.792493 | 0.000367 |
| EIF2A | 0.654352 | 0.527277 | 0.812052 | 0.000118 |
| EPB41L2 | 0.729634 | 0.614815 | 0.865896 | 0.000308 |
| EPPK1 | 1.933011 | 1.316561 | 2.8381 | 0.00077 |
| FAM83H | 1.371429 | 1.173052 | 1.603353 | 7.43E-05 |
| FGF2 | 0.73005 | 0.616223 | 0.864903 | 0.000275 |
| FNDC3B | 0.686523 | 0.565361 | 0.833652 | 0.000147 |
| GABARAPL2 | 0.606555 | 0.454116 | 0.810164 | 0.00071 |
| GBP2 | 0.716804 | 0.649896 | 0.790602 | 2.75E-11 |
| GCA | 0.597119 | 0.483436 | 0.737536 | 1.71E-06 |
| GIMAP5 | 0.558735 | 0.422148 | 0.739516 | 4.70E-05 |
| GOLGB1 | 0.672137 | 0.539433 | 0.837487 | 0.0004 |
| GOT2 | 1.563622 | 1.221521 | 2.001532 | 0.000388 |
| GPI | 1.485826 | 1.21583 | 1.815779 | 0.000109 |
| GRB7 | 1.736016 | 1.286415 | 2.342752 | 0.00031 |
| GRWD1 | 1.868894 | 1.392356 | 2.508528 | 3.13E-05 |
| GSTP1 | 1.224391 | 1.092698 | 1.371955 | 0.000489 |
| HDAC8 | 0.438733 | 0.282032 | 0.682498 | 0.000258 |
| HMOX2 | 2.07067 | 1.487736 | 2.882013 | 1.60E-05 |
| IBTK | 0.681992 | 0.547162 | 0.850045 | 0.00066 |
| IQCB1 | 0.601301 | 0.474689 | 0.761683 | 2.48E-05 |
| ITK | 0.691467 | 0.576034 | 0.830033 | 7.53E-05 |
| KCTD6 | 0.456239 | 0.31562 | 0.659509 | 2.99E-05 |
| KHDRBS3 | 0.751468 | 0.653881 | 0.863619 | 5.68E-05 |
| KIT | 1.178028 | 1.102579 | 1.25864 | 1.23E-06 |
| KRT1 | 1.120235 | 1.056301 | 1.188039 | 0.000153 |
| KRT17 | 1.155862 | 1.094544 | 1.220614 | 1.91E-07 |
| LAP3 | 0.625752 | 0.533964 | 0.733319 | 6.94E-09 |
| LARP7 | 0.69431 | 0.569391 | 0.846634 | 0.000312 |
| LCK | 0.764933 | 0.67811 | 0.862873 | 1.30E-05 |
| LCP1 | 0.829662 | 0.75963 | 0.906151 | 3.32E-05 |
| LGALS7B | 1.163287 | 1.078382 | 1.254878 | 9.17E-05 |
| LRRK2 | 0.628803 | 0.50422 | 0.784168 | 3.82E-05 |
| LYZ | 0.86177 | 0.803337 | 0.924453 | 3.29E-05 |
| MAD1L1 | 1.363808 | 1.150127 | 1.61719 | 0.000359 |
| MBNL1 | 0.663316 | 0.554971 | 0.792814 | 6.44E-06 |
| MED10 | 0.599824 | 0.44893 | 0.801435 | 0.000546 |
| MLKL | 0.558117 | 0.442251 | 0.70434 | 9.01E-07 |
| MTUS1 | 0.768294 | 0.658593 | 0.896267 | 0.000799 |
| MYO1F | 0.668442 | 0.562186 | 0.794782 | 5.11E-06 |
| NDRG1 | 0.833609 | 0.754053 | 0.921559 | 0.000376 |
| NEK7 | 0.714122 | 0.600809 | 0.848807 | 0.000134 |
| NR3C1 | 0.67462 | 0.549874 | 0.827666 | 0.000161 |
| OAS1 | 0.771005 | 0.680871 | 0.873072 | 4.13E-05 |
| OAS2 | 0.826736 | 0.745887 | 0.916349 | 0.00029 |
| OFD1 | 0.638513 | 0.501679 | 0.812669 | 0.000267 |
| PAK4 | 1.696292 | 1.330976 | 2.161877 | 1.95E-05 |
| PARP12 | 0.658168 | 0.568858 | 0.761501 | 1.89E-08 |
| PARVA | 1.490757 | 1.19475 | 1.860102 | 0.000407 |
| PIK3R2 | 2.129646 | 1.449983 | 3.127892 | 0.000116 |
| PIK3R6 | 0.540598 | 0.395826 | 0.738319 | 0.00011 |
| PKP1 | 1.230354 | 1.131864 | 1.337413 | 1.12E-06 |
| PNRC1 | 0.601103 | 0.466716 | 0.774186 | 8.07E-05 |
| POU2AF1 | 0.786921 | 0.687265 | 0.901028 | 0.000523 |
| PPA1 | 0.664041 | 0.53186 | 0.829074 | 0.0003 |
| PPP1R2 | 0.623732 | 0.494229 | 0.787168 | 7.02E-05 |
| PPP4R2 | 0.684626 | 0.548022 | 0.855279 | 0.000848 |
| PRKAR2B | 0.701485 | 0.593846 | 0.828635 | 3.02E-05 |
| PRKCQ | 0.674269 | 0.545645 | 0.833214 | 0.000263 |
| PSMA3 | 0.571026 | 0.436472 | 0.74706 | 4.37E-05 |
| PSMA4 | 0.602759 | 0.463075 | 0.784576 | 0.000167 |
| PSMB10 | 0.773644 | 0.673773 | 0.888319 | 0.000273 |
| PSME1 | 0.584572 | 0.475377 | 0.718849 | 3.60E-07 |
| PSME2 | 0.620617 | 0.51302 | 0.75078 | 9.08E-07 |
| PTK2B | 0.647423 | 0.524818 | 0.798669 | 4.94E-05 |
| PYHIN1 | 0.678093 | 0.579712 | 0.793171 | 1.19E-06 |
| RAB8B | 0.697437 | 0.57838 | 0.841002 | 0.000161 |
| RAD21 | 0.730099 | 0.60753 | 0.877396 | 0.000794 |
| RANBP6 | 0.590776 | 0.45989 | 0.758912 | 3.81E-05 |
| RASSF5 | 0.7604 | 0.656163 | 0.881195 | 0.000271 |
| RBM27 | 0.588695 | 0.43305 | 0.80028 | 0.000719 |
| REC8 | 0.56864 | 0.44133 | 0.732675 | 1.27E-05 |
| RNASET2 | 0.698923 | 0.582086 | 0.839211 | 0.000124 |
| RNF114 | 0.528931 | 0.397421 | 0.703958 | 1.26E-05 |
| RNF19A | 0.690603 | 0.565456 | 0.843449 | 0.000285 |
| RNF213 | 0.605802 | 0.490109 | 0.748805 | 3.56E-06 |
| RPF1 | 0.622518 | 0.483237 | 0.801943 | 0.000244 |
| RPGR | 0.531045 | 0.39806 | 0.708459 | 1.68E-05 |
| RPP25 | 1.301656 | 1.145419 | 1.479204 | 5.32E-05 |
| RPS6KA3 | 0.720427 | 0.597332 | 0.868889 | 0.000603 |
| S100A7A | 1.366844 | 1.208561 | 1.545859 | 6.47E-07 |
| SACM1L | 0.658713 | 0.515679 | 0.84142 | 0.000831 |
| SBSN | 1.158631 | 1.083468 | 1.239009 | 1.69E-05 |
| SCFD1 | 0.573958 | 0.434962 | 0.757372 | 8.70E-05 |
| SERPINB3 | 1.261758 | 1.129924 | 1.408974 | 3.64E-05 |
| SFN | 1.138481 | 1.072814 | 1.208166 | 1.88E-05 |
| SLC25A3 | 1.87994 | 1.369574 | 2.580491 | 9.38E-05 |
| SMAP2 | 0.697415 | 0.568249 | 0.855941 | 0.000564 |
| SMARCA4 | 1.62177 | 1.246966 | 2.109228 | 0.000311 |
| SNRPB2 | 0.675128 | 0.547745 | 0.832135 | 0.000231 |
| SOCS1 | 0.724519 | 0.617099 | 0.850639 | 8.30E-05 |
| SP110 | 0.625757 | 0.484382 | 0.808395 | 0.000333 |
| SPATC1 | 0.077304 | 0.021674 | 0.275711 | 7.95E-05 |
| SPINK7 | 1.51464 | 1.220543 | 1.879601 | 0.000164 |
| SPOPL | 0.609394 | 0.46398 | 0.800382 | 0.00037 |
| SPTY2D1 | 0.607766 | 0.462525 | 0.798616 | 0.000352 |
| STAT1 | 0.730189 | 0.653232 | 0.816213 | 3.13E-08 |
| STXBP3 | 0.671966 | 0.530464 | 0.851214 | 0.000983 |
| TAP1 | 0.798601 | 0.71909 | 0.886902 | 2.63E-05 |
| TCEA1 | 0.628658 | 0.4948 | 0.798728 | 0.000145 |
| THYN1 | 0.507603 | 0.381965 | 0.674565 | 2.96E-06 |
| TOMM40 | 1.532033 | 1.194986 | 1.964144 | 0.000765 |
| TRIM21 | 0.658369 | 0.547336 | 0.791926 | 9.19E-06 |
| TRIM22 | 0.709893 | 0.627939 | 0.802542 | 4.39E-08 |
| TRIM56 | 0.589187 | 0.465309 | 0.746044 | 1.12E-05 |
| TROAP | 1.391237 | 1.143011 | 1.693369 | 0.000991 |
| TSG101 | 0.57504 | 0.414947 | 0.7969 | 0.000888 |
| TUBA1C | 1.474644 | 1.204922 | 1.804744 | 0.000164 |
| UBXN4 | 0.668298 | 0.550679 | 0.811039 | 4.49E-05 |
| USP33 | 0.646711 | 0.517123 | 0.808774 | 0.000133 |
| XIAP | 0.586199 | 0.456191 | 0.753258 | 2.98E-05 |
| XRN1 | 0.568252 | 0.456325 | 0.707633 | 4.42E-07 |
| YPEL5 | 0.646746 | 0.507632 | 0.823984 | 0.000421 |
| ZBP1 | 0.563895 | 0.44449 | 0.715375 | 2.37E-06 |
| ZBTB1 | 0.65823 | 0.515695 | 0.840162 | 0.000783 |
| ZCRB1 | 0.642088 | 0.50899 | 0.809992 | 0.000186 |
| ZNF277 | 0.632077 | 0.497543 | 0.802989 | 0.000172 |

**Table S3B. The coefficients evaluated by multivariate Cox regression**

| **Id** | **coef** | **HR** | **HR.95L** | **HR.95H** | ***p*-value** |
| --- | --- | --- | --- | --- | --- |
| MLKL | -2.3290 | 0.0974 | 0.0290 | 0.3270 | 0.0002 |
| PARVA | 1.6786 | 5.3578 | 1.2475 | 23.0117 | 0.0240 |
| PKP1 | 0.5818 | 1.7892 | 1.1411 | 2.8052 | 0.0112 |
| PSME1 | -3.0506 | 0.0473 | 0.0054 | 0.4147 | 0.0059 |
| RNF114 | -2.0959 | 0.1230 | 0.0156 | 0.9667 | 0.0464 |
| TROAP | 1.8475 | 6.3438 | 2.1248 | 18.9399 | 0.0009 |
