# Supplementary material for: Liquid-liquid phase separation throws novel insights into treatment strategies for skin cutaneous melanoma
Source: BMC Cancer. 2023 May 1;23:388. doi: 10.1186/s12885-023-10847-w (PMC10150491; doi:10.1186/s12885-023-10847-w)
Supplement: Supplementary file 1 — Additional file 1. [file 12885_2023_10847_MOESM1_ESM.zip › Supplementary file/Supplementary Figures.docx]

**
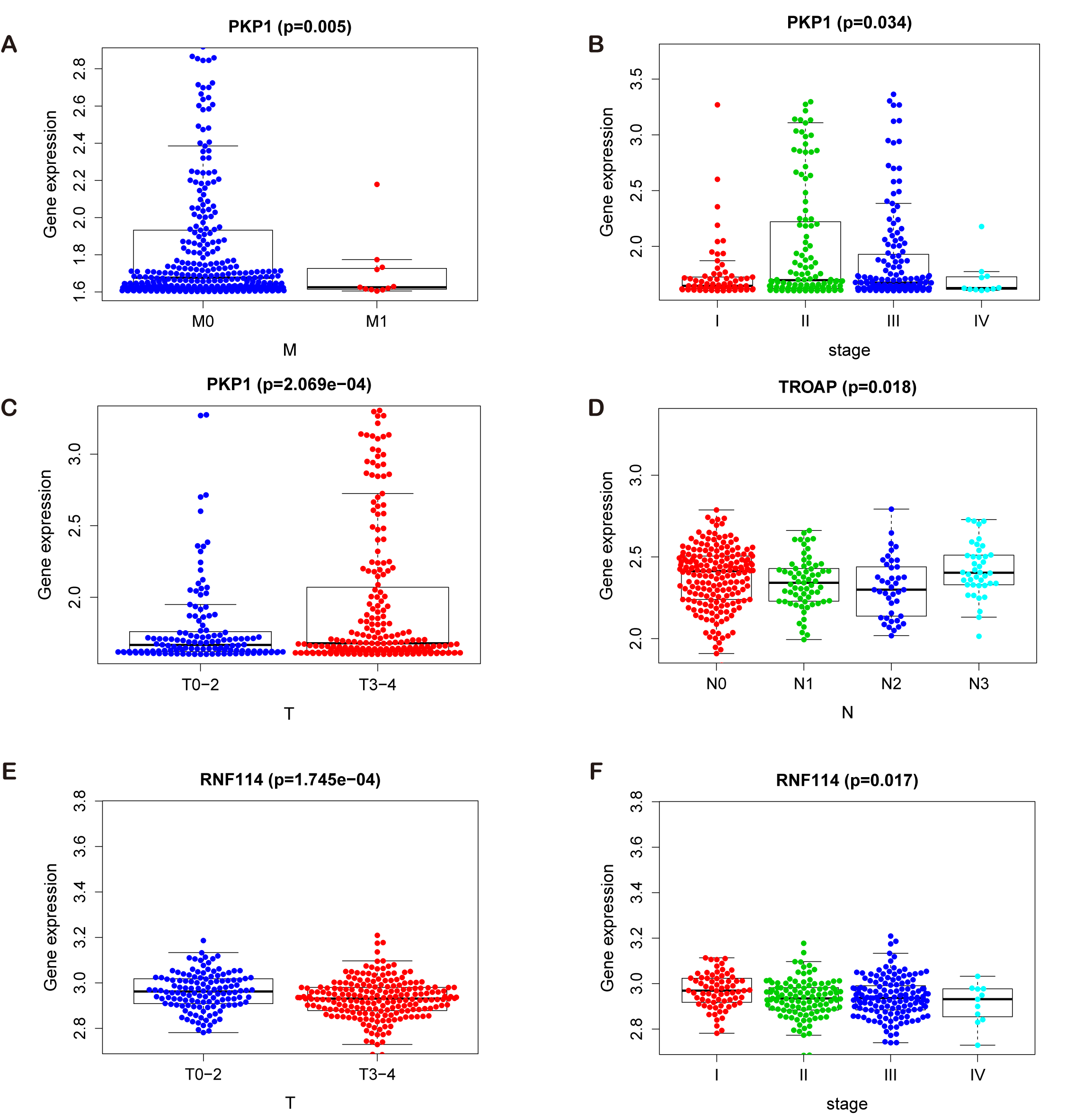
**

**Figure S1** Relationship between candidate gene expression levels and clinicopathological features. RKP1 was highly expressed in M0 (A), stage II/III (B), and T3-4 (C) melanoma patients. (D)There were significant differences in the expression of TROAP in melanoma patients with different N stages. RNF114 expression was significantly different in melanoma patients with different T classifications (E) and stages (F).


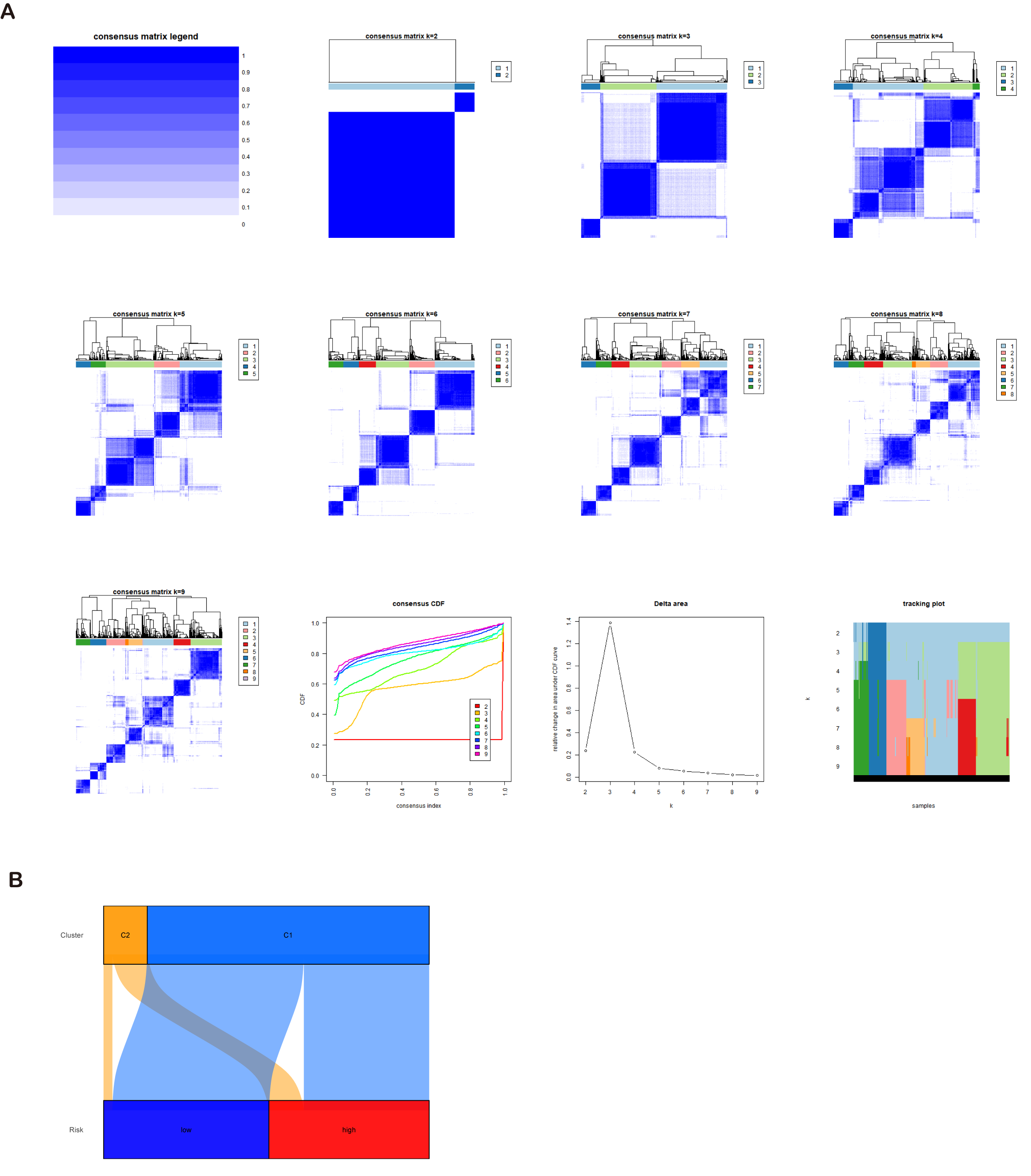


**Figure S2** The detailed process of tumor typing and the relationship between each subtype and risk scores. (A) Consensus clustering analysis process for risk genes. (B) Cluster distribution of high-risk and low-risk groups, with cluster 2 patients mainly belonging to the high-risk group.


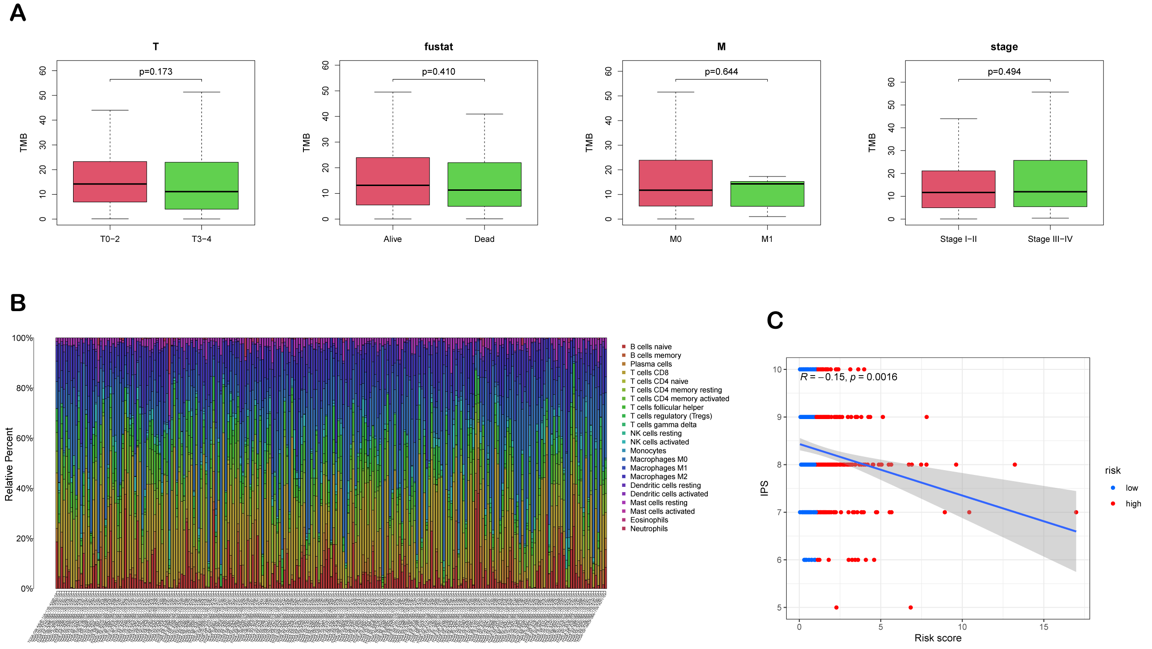


**Figure S3** The tumor mutation burden, immune infiltration, IPS of melanoma patients. (A) No significant differences in TMB values were observed in the clinical subgroup analysis (T, fustat, M, stage). (B) The bar plot demonstrated the relative proportions of 22 infiltrating immune cells represented by various colors in melanoma patients. (C) IPS score was significantly negatively correlated with risk score (p = 0.0016).


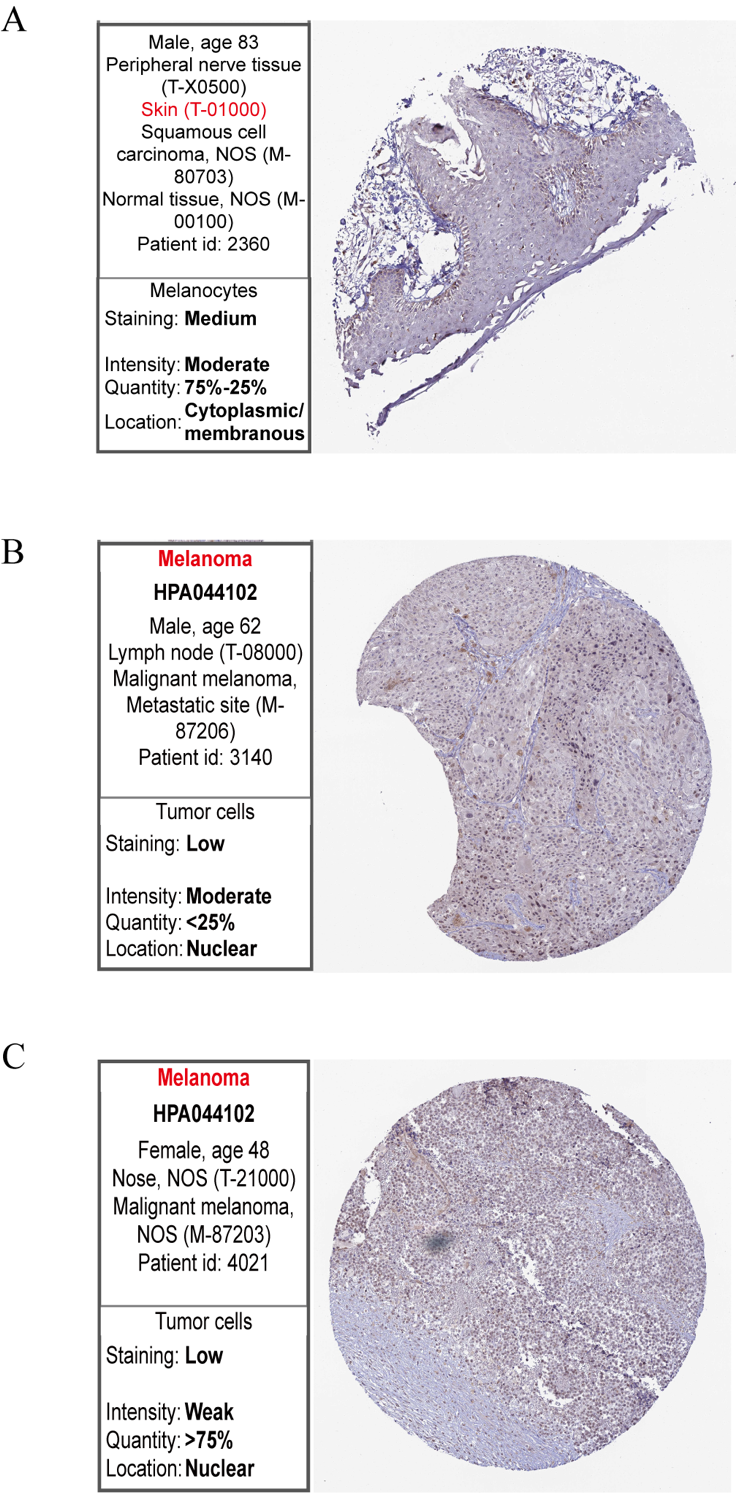


**Figure S4** Comparison of relative TROAP expression between melanoma and normal skin tissues in The Human Protein Atlas database. (A) Immunohistochemistry of TROAP expression in the tissue microarray of normal skin tissues (Scale bar, 200 μm). (B, C) Immunohistochemical staining of TROAP in melanoma (Scale bar, 200 μm).
