# Supplementary material for: Liquid-liquid phase separation throws novel insights into treatment strategies for skin cutaneous melanoma
Source: BMC Cancer. 2023 May 1;23:388. doi: 10.1186/s12885-023-10847-w (PMC10150491; doi:10.1186/s12885-023-10847-w)
Supplement: Supplementary file 1 — Additional file 1. [file 12885_2023_10847_MOESM1_ESM.zip › Supplementary file/Table S2.docx]

**Table S2. Characteristics of different cohorts of melanoma patients**

|  | **No. (%)** | | | |
| --- | --- | --- | --- | --- |
| **Characteristics** | **Entire**  **cohort**  **(n=447)** | **Training**  **group**  **(n=224)** | **Testing**  **group**  **(n=223)** | ***P* value** |
| **Age** |  |  |  | 0.263 |
| <65 | 286 (64.0) | 149 (66.5) | 137 (61.4) |  |
| >=65 | 161(36.0) | 75 (33.5) | 86 (38.6) |  |
| **Gender** |  |  |  | 0.311 |
| Female | 168 (37.6) | 79 (35.3) | 89 (39.9) |  |
| Male | 279 (62.4) | 145 (64.7) | 134 (60.1) |  |
| **Fustat** |  |  |  | 0.422 |
| Alive | 229 (51.2) | 119 (53.1) | 110 (49.3) |  |
| Died | 218 (48.8) | 105 (46.9) | 113 (50.7) |  |
| **Stage** |  |  |  | 0.261 |
| Stage I | 76 (17.0) | 39 (17.4) | 37 (16.6) |  |
| Stage II | 133 (29.8) | 72 (32.1) | 61 (27.4) |  |
| Stage III | 169 (37.8) | 83 (37.1) | 86 (38.6) |  |
| Stage IV | 20 (4.5) | 12 (5.4) | 8 (3.6) |  |
| Unknow | 49 (11.0) | 18 (8.0) | 31 (13.9) |  |
| **T** |  |  |  | 0.069 |
| T0 | 23 (5.1) | 15 (6.7) | 8 (3.6) |  |
| T1 | 41 (9.2) | 24 (10.7) | 17 (7.6) |  |
| T2 | 76 (17.0) | 35 (15.6) | 41 (18.4) |  |
| T3 | 89 (19.9) | 49 (21.9) | 40 (17.9) |  |
| T4 | 144 (32.2) | 74 (33.0) | 70 (31.4) |  |
| unknow | 74 (16.6) | 27 (12.1) | 47 (21.1) |  |
| **M** |  |  |  | 0.128 |
| M0 | 402 (89.9) | 206 (92.0) | 196 (87.9) |  |
| M1 | 24 (5.4) | 12 (5.4) | 12 (5.4) |  |
| unknow | 21 (4.7) | 6 (2.7) | 15 (6.7) |  |
| **N** |  |  |  | 0.479 |
| N0 | 222 (49.7) | 120 (53.6) | 102 (45.7) |  |
| N1 | 73 (16.3) | 35 (15.6) | 38 (17.0) |  |
| N2 | 49 (11.0) | 23 (10.3) | 26 (11.7) |  |
| N3 | 54 (12.1) | 26 (11.6) | 28 (12.6) |  |
| unknow | 49 (11.0) | 20 (8.9) | 20 (8.9) |  |
