# Supplementary material for: Liquid-liquid phase separation throws novel insights into treatment strategies for skin cutaneous melanoma
Source: BMC Cancer. 2023 May 1;23:388. doi: 10.1186/s12885-023-10847-w (PMC10150491; doi:10.1186/s12885-023-10847-w)
Supplement: Supplementary file 1 — Additional file 1. [file 12885_2023_10847_MOESM1_ESM.zip › Supplementary file/Table S1.docx]

**Table S1. Oligonucleotides used in research**

| **Oligonucleotides** | **Nucleotide sequence (5'-3')** |
| --- | --- |
| **shRNA** |  |
| scramble control | GCUUCGCGCCGUAGUCUUA |
| shTROAP-1 | GGAGAGUUGUAUAAGGUCACU |
| shTROAP-2 | GTAGGATTGAGCCTGAGAT |
| **Primer** |  |
| GAPDH | GGCCTCCAAGGAGTAAGACC (forward) |
|  | AGGGGAGATTCAGTGTGGTG (reverse) |
| TROAP | GGCAGGCCTCAGCAATCTG (forward) |
|  | GGCATCCTGCCATTCGAGTA (reverse) |
